# Emerging Roles for Sphingolipids in Cardiometabolic Disease: A Rational Therapeutic Target?

**DOI:** 10.3390/nu16193296

**Published:** 2024-09-28

**Authors:** Daniel Foran, Charalambos Antoniades, Ioannis Akoumianakis

**Affiliations:** Cardiovascular Medicine Division, Radcliffe Department of Medicine, University of Oxford, Oxford OX3 9DU, UK; daniel.foran@cardiov.ox.ac.uk (D.F.); charalambos.antoniades@cardiov.ox.ac.uk (C.A.)

**Keywords:** sphingolipid, ceramide, sphingomyelin, cardiovascular disease, atherosclerosis, heart failure, inflammation

## Abstract

Cardiovascular disease is a leading cause of morbidity and mortality. New research elucidates increasingly complex relationships between cardiac and metabolic health, giving rise to new possible therapeutic targets. Sphingolipids are a heterogeneous class of bioactive lipids with critical roles in normal human physiology. They have also been shown to play both protective and deleterious roles in the pathogenesis of cardiovascular disease. Ceramides are implicated in dysregulating insulin signalling, vascular endothelial function, inflammation, oxidative stress, and lipoprotein aggregation, thereby promoting atherosclerosis and vascular disease. Ceramides also advance myocardial disease by enhancing pathological cardiac remodelling and cardiomyocyte death. Glucosylceramides similarly contribute to insulin resistance and vascular inflammation, thus playing a role in atherogenesis and cardiometabolic dysfunction. Sphingosing-1-phosphate, on the other hand, may ameliorate some of the pathological functions of ceramide by protecting endothelial barrier integrity and promoting cell survival. Sphingosine-1-phosphate is, however, implicated in the development of cardiac fibrosis. This review will explore the roles of sphingolipids in vascular, cardiac, and metabolic pathologies and will evaluate the therapeutic potential in targeting sphingolipids with the aim of prevention and reversal of cardiovascular disease in order to improve long-term cardiovascular outcomes.

## 1. Introduction

Cardiovascular disease (CVD) remains the leading cause of death worldwide [1]. Metabolic pathogenic processes such as insulin resistance and obesity are increasingly linked with CVD pathogenesis; however, the underlying mechanisms remain incompletely characterised [2]. With an ageing population and increasing prevalence of type 2 diabetes mellitus (T2DM) and obesity, the global burden of cardiovascular disease is expected to increase [1]. This highlights an unmet need to discover novel disease mediators and develop effective therapies for cardiometabolic disease.

Sphingolipids are a heterogeneous class of bioactive lipids, increasingly associated with cardiovascular disease [3]. Initially discovered as an integral structural component of cell membranes, research over recent decades has uncovered both protective and detrimental roles for sphingolipids in relation to cardiovascular health as well as their potential as therapeutic targets [4]. However, current research work has failed to translate into clinically relevant applications. This narrative review will summarise the important and recent evidence pertaining to the role and putative mechanisms of sphingolipids in CVD and the different potential approaches to treat pathological sphingolipid signalling and metabolism.

## 2. Sphingolipids: Fundamentals of Biology and Metabolism

Sphingolipids are a family of complex lipids featuring a long-chain amino alcohol, known as a sphingoid base, as their defining component [5]. More complex sphingolipids may comprise a sphingoid base bound to a fatty acid via an amide bond [6]. Sphingolipids can be differentiated by their many different functional head groups [6]. Sphingoid bases are the simplest sphingolipids and can be modified and metabolised into a range of more complex derivatives including ceramides, sphingomyelins, glycosphingolipids, and phosphorylated sphingolipids [7].

### 2.1. Sphingolipid Biosynthesis, Metabolism, and Catabolism

Simple and common sphingolipids are generally synthesised in all cells, with biosynthesis primarily localised to the cytosolic membrane of the endoplasmic reticulum (ER) and Golgi body [8]. However, biosynthesis of more complex sphingolipids may in some cases be relatively enriched in specific cell types compared to others, depending on the function of the product [9]. Furthermore, the amphipathicity of different sphingolipid species determines the localisation of the biosynthetic processes a given species is involved in [7]. Sphingosine and dihydrosphingosine are amphithetic enough to traverse membranes, meaning that most relevant enzymatic reactions occur on the cytosolic membrane of the ER [7]. Ceramides, sphingomyelins, and glycosphingolipids on the other hand have low amphipathicity, meaning they are mostly modified enzymatically in the luminal membranes of the ER or Golgi [7]. Sphingosine-1-phosphate (S1P) and ceramide-1-phosphate (C1P) are hydrophilic and can roam hydrophilic compartments such as the cytosol; hence, the relevant enzymes possess a cytosolic binding site [7].

**Figure 1:**
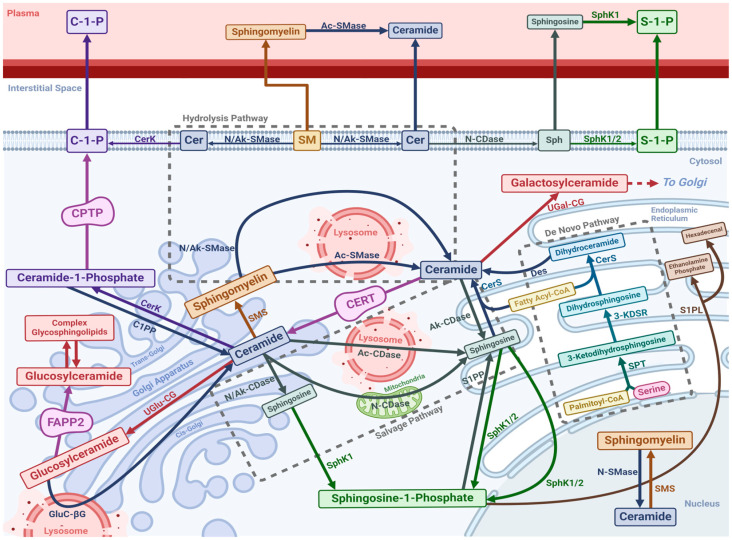
A diagram exhibiting the sphingolipid biosynthetic, metabolic, and catabolic networks including the intracellular organelles and extracellular compartments in which the individual reactions occur. Highlighted are the three pathways contributing to ceramide synthesis: the de novo, hydrolysis, and salvage pathways. SPT: Serine Palmitoyl Transferase, 3-KDSR: 3-Ketodihydrosphingosine Reductase, CerS: Ceramide Synthase, Des: Dihydroceramide Desaturase, CERT: Ceramide Endoplasmic Reticulum Transport Protein, SMS: Sphingomyelin Synthase, Ac-SMase: Acid Sphingomyelinase, N-SMase: Neutral Sphingomyelinase, N/Ak-SMase: Neutral/Alkaline Sphingomyelinase, Ac-CDase: Acid Ceramidase, N-CDase: Neutral Ceramidase, Ak-CDase: Alkaline Ceramidase, SphK: Sphingosine Kinase, S1PP: Sphingosine-1-Phosphate Phosphatase, S1PL: Sphingosine-1-Phosphate Lyase, UGal-CG: UDP Galactose Ceramide Galactosyltransferase, UGlu-CG: UDP Glucose Ceramide Glucosyltransferase, GluC-βG: Glucosylceramide β-Glucosidase, FAPP2: Four-Phosphate Adaptor Protein 2, CerK: Ceramide Kinase, C1PP: Ceramide-1-Phosphate Phosphatase, CPTP: Ceramide-1-Phosphate Transport Protein, C-1-P: Ceramide-1-Phosphate, Cer: Ceramide, SM: Sphingomyelin, Sph: Sphingosine, S-1-P: Sphingosine-1-Phosphate, P: Phosphate, Pc: Phosphocholine. Different colours distinguish the unique products of individual enzymatic processes (for example arrows and enzymes that directly lead to ceramide generation are in dark blue etc.). Created with BioRender.com.

Sphingolipids are all linked in a web of synthesis, modification, metabolism and salvage (Figure 1) but with only a single entry and exit point into and out of the cycle [10]. The de novo synthesis pathway represents the single entry point of sphingolipid constituents into the metabolic cycle, whilst S1P lyase (SPL) represents the single exit pathway [10].

#### 2.1.1. Sphingoid Bases

Sphingoid bases are the simplest of sphingolipids [11]. Though they form the basis for the synthesis of more complex derivatives, they too have their own unique and important functions relating to cytotoxicity and apoptosis [12]. The most ubiquitous sphingoid bases include sphingosine, dihydrosphingosine (also known as sphinganine) and hydroxysphingosine (or phytosphingosine) [12,13]. Minor variations to these bases give rise to over 60 structurally different sphingoid bases [13]. Sphingoid bases are structurally long-chain, amino alcohols [13], with 18-carbon sphingoid bases predominating in humans [14]. Other chain lengths from 12 up to 26 carbons are also possible, albeit less common [15].

Unlike dihydrosphingosine, sphingosine is not typically synthesised de novo in eukaryotes, with the only sources of production being hydrolysis of ceramide or exogenous delivery [12]. Dihydrosphingosine, contrastingly, is the molecular product of the second step in the de novo ceramide synthesis pathway, resulting from the combination of palmitoyl co-enzyme A (CoA) and l-serine and subsequent reduction [16]. The three core sphingoid bases mentioned above may undergo subsequent modification to create sphingoid derivatives [11]. Phosphorylation of the terminal hydroxyl group present throughout all sphingoid bases, for example, would give rise to S1P [11].

#### 2.1.2. Ceramides

Ceramide comprises a sphingoid base combined with a long-chain fatty acid [17]. Ceramide acts as the nexus of the sphingolipid metabolic network, from which almost all other species are derived [18]. Three pathways contribute to ceramide biosynthesis: the de novo synthesis pathway, the hydrolytic pathway, and the salvage pathway (Figure 1) [19].

De novo ceramide synthesis occurs on the cytosolic membrane of the ER [7]. Serine palmitoyltransferase (SPT) initiates the process by converting palmitoyl-CoA and serine into 3-Ketodihydrosphingosine [5,20], which is then reduced to dihydrosphingosine [5,20]. Dihydrosphingosine is acylated with a fatty acid chain by one of six ceramide synthases (CerS), creating dihydroceramide [5,20]. Each CerS variety has its own fatty acyl CoA substrate chain-length preference and will, as a result, produce a distinct ceramide species [21]. Finally, desaturase desaturates dihydroceramide by introducing a trans double bond to form ceramide [22]. Ceramide is then transported from the ER to the Golgi apparatus via either the ceramide ER transport protein or a separate vesicular transport process [23]. In the Golgi, ceramide may then be used to generate more complex sphingolipids such as sphingomyelin or glycosphingolipids [24].

Ceramide can also be generated via catabolism of sphingomyelin and other complex sphingolipids, via catabolic enzymes called sphingomyelinases (SMases) [19]. Three core varieties of SMase exist, including acid (Ac-SMase), alkaline (Ak-SMase), and neutral (N-SMase) SMases [7]. Ac-SMase is primarily expressed in the lysosomal compartment [7], whilst a secretory form of Ac-SMase also exists, which can deconstruct plasma sphingomyelin [25]. Ak-SMase and N-SMase are expressed in the Golgi and the plasma membrane [7,25]. Oxidative stress and inflammation are able to upregulate SMase activity resulting in cellular accumulation of ceramides [26]. In addition to sphingomyelin hydrolysis, glycosphingolipid deconstruction can also yield ceramide [25]. Typically, all glycosphingolipids are deconstructed back to either glucosylceramide or galactosylceramide which are then hydrolysed by glucosylceramide-β-glucosidase and galactosylceramide-β-galactosidase, respectively, to regenerate ceramide [25]. Both enzymes are primarily lysosomal [27].

Ceramides are catabolised by a class of enzymes known as ceramidases (CDases) [25], which can be found in at least five variants [5]. The three main variants include acid (Ac-CDase), alkaline (Ak-CDase), and neutral (N-CDase) CDases, all of which deacylate ceramide back to sphingosine [25]. Whereas Ac-CDase is primarily localised to the lysosomal compartment, Ak-CDase is found in the ER and Golgi and N-CDase in the Golgi, mitochondria, and plasma membrane [25]. This salvage pathway (Figure 1) allows the sphingosine generated from ceramide degradation to be recycled into new sphingolipids [28].

#### 2.1.3. Sphingomyelin

Sphingomyelin is the most abundant sphingolipid in the human body [7], originating from ceramide by the addition of a phosphocholine headgroup [29]. This is catalysed by sphingomyelin synthase (SMS) enzymes, primarily on the luminal Golgi membrane [18]. A second SMS variant localised in the plasma membrane with an active site facing the extracellular space has also been described [30], which may be responsible for maintaining sufficient plasma membrane sphingomyelin levels [7]. As with most sphingolipids, distinct individual sphingomyelins arise as a result of ceramide acyl chain variation rather than the phosphocholine group. As described above, sphingomyelin is converted back into ceramide by SMases [31].

#### 2.1.4. Glycosphingolipids

Glycosphingolipids are ceramide derivatives distinguished by their complex carbohydrate headgroups; they represent the most complex sphingolipids [32]. There are two main subclasses of glycosphingolipids known as glucosphingolipids and galactosphingolipids [7]. Glucosphingolipids arise from glucosylceramide, comprising merely ceramide and glucose [27]. Galactosphingolipids arise from galactosylceramide, a combination of ceramide and galactose [32]. Glucosylceramide and galactosylceramide are both generated via uridine diphosphate (UDP) glycosyltransferase enzymes; UDP-glucose ceramide glucosyltransferase (UGlu-CG) generates glucosylceramide and UDP-galactose ceramide galactosyltransferase (UGal-CG) generates galactosylceramide [9]. UGlu-CG synthesises glucosylceramide in the cytosolic membrane of the cis-Golgi [5]. Glucosylceramide is then transported to the luminal compartment of the trans-Golgi by the four-phosphate adaptor protein 2 [33]. Conversely, UGal-CG generates galactosylceramide in the ER [34]. Once glucosylceramide and galactosylceramide have been synthesised, more complex glycosphingolipids are generated via a large family of glycosyltransferases, primarily in the luminal compartment of the Golgi [33].

#### 2.1.5. Ceramide-1-Phosphate

C1P is a phosphosphingolipid, generated as a result of the phosphorylation of ceramide by the ceramide kinase (CerK) enzyme [20]. Whilst CerK is a cytosolic enzyme, it primarily catalyses ceramide phosphorylation on the cytosolic trans-Golgi but also on the plasma membranes [35]. Once generated, C1P may be transported to the plasma membrane or other intracellular organelles by the ceramide-1-phosphate transport protein to function as a signalling molecule [36]. More recent evidence also suggests that C1P can be released into the extracellular space and act on extracellular receptors [37]. C1P can then be dephosphorylated back into ceramide intracellularly; however, this catabolic pathway is less well defined than those of other sphingolipids [7]. Some evidence from animal studies suggests that specific C1P phosphatase (C1PP) activity is present in both brain and liver tissue at the level of the plasma membrane [38]. However, other research demonstrates that a family of non-specific phosphatases known as lipid phosphate phosphatases can also de-phosphorylate C1P and S1P amongst other lipid-phosphate complexes [39].

#### 2.1.6. Sphingosine-1-Phosphate

S1P is perhaps the most studied sphingolipid to date and is a critical signalling molecule in multiple metabolic processes [40]. S1P is formed by the addition of a phosphate headgroup to a sphingosine moiety [41]. There appear to be two distinct sphingosine kinase (SphK) isoforms responsible for the synthesis of S1P: SphK1 and SphK2 [42]. Both are cytosolic enzymes but demonstrate differences in localisation [42]. Whereas SphK1 is localised primarily to the cytoplasm with expression also in the ER, nucleus, plasma membrane, and circulating plasma, SphK2 tends to localise preferentially to the nucleus [43]. The fact that SphK is present in the circulation suggests a role for secretory S1P that is generated after sphingosine has been released from cells [44]. Once synthesised, S1P may either translocate to other cellular organelles or be secreted into the extracellular space [40]. Whilst the mechanisms behind S1P extracellular secretion are incompletely understood, evidence from research on activated platelets implicates protein kinase C (PKC) binding in the process [45].

There are two possible catabolic destinies for S1P. The first involves S1P being dephosphorylated back to sphingosine by S1P phosphatases (S1PP) at the cytosolic membrane of the ER [11]. Other non-specific phosphatases such as LPP1, 2, and 3 have also been shown to catalyse the dephosphorylation of S1P back to sphingosine [46]. The second pathway represents an exit of the constituent components of sphingolipids from the sphingolipid metabolic network. S1P lyase, a transmembrane protein exclusive to the cytosolic membrane of the ER, degrades S1P to hexadecenal and phosphoethanolamine in the ER, thus ending the sphingolipid life cycle [47].

### 2.2. Physiological Roles of Sphingolipids

Sphingolipids are integral to a broad array of physiological functions including cell membrane function and integrity, innate and adaptive immune modulation, cell cycle and apoptosis regulation, responses to stress and inflammation, cell adhesion and migration, and inter/intracellular signalling amongst many others. The following sections highlight some of the most well-characterised, though non-exhaustive, sphingolipid functions.

#### 2.2.1. Sphingoid Bases

Sphingoid bases such as sphingosine are vital to a number of physiological processes. One of their chief functions is the modulation of cell plasma membrane properties [48]. Sphingosine appears to promote plasma membrane fluidity by virtue of its existence as a gel at a physiological body temperature [48]. Sphingosine’s integration into lipid membranes may be able to tighten membrane intermolecular packing, thereby increasing membrane stability and promoting the formation of ordered domains such as lipid rafts [49]. Low levels of protonated sphingosine can further enhance phospholipid membrane stability through the use of hydrogen bonding and electrostatic interactions to tighten the phospholipid packing [49]. Sphingosine is also present in blood at concentrations of 0.1 to 1 μMol/L and may demonstrate micellar behaviours potentially capable of solubilising membranes and membrane proteins [12]. Increasing membrane sphingolipid concentrations may have a physiological purpose, however, as they can increase plasma membrane permeability [50]. To this end, sphingosine may be involved in positively regulating endocytic membrane trafficking by promoting endocytic vesicle formation and survival [51].

Sphingosine also modulates intracellular signalling [48]. Sphingosine can both directly and indirectly inhibit PKC [52], which has multiple downstream effects, often involving cardiometabolic disease [53]. Positive outcomes of sphingosine-mediated PKC inhibition include vasorelaxation and reduced platelet aggregation, whilst deleterious phenotype changes include impaired insulin signalling and a negative inotropic effect in the myocardium [53]. PKC also modulates sphingolipid metabolism as PKC is essential for Ac-SMase activation [54]. Thus, sphingosine may indirectly downregulate Ac-SMase activity and, as a result, ceramide generation from sphingomyelin [54]. Sphingosine also modulates the activity of other protein kinases including calmodulin-dependent kinase, casein kinase fam20c, and the insulin receptor (IR) tyrosine kinase [55]. The case of the latter highlights a potential role for sphingosine in the pathophysiology behind developing insulin resistance and metabolic dysfunction [56]. Indeed, PKC’s purported role in dysregulating insulin signalling through inhibition of the phosphatidylinositol 3-kinase (PI3K)/Akt pathway may suggest a role for sphingosine in ameliorating cellular insulin resistance phenotypes [57]. Sphingosine’s power over cell signalling kinases also allows it to promote and regulate cell growth arrest and apoptosis [58].

#### 2.2.2. Ceramides

Ceramides boast a range of physiological functions including negative regulation of cellular nutrient uptake, cell proliferation and survival, induction of cell growth arrest and apoptosis, and as a critical component of lipid membrane structures in the skin amongst many others [5].

Ceramide is also crucial in nutrient handling. Indeed, it seems to act as an antagonistic force against intracellular insulin signalling and inhibits insulin-mediated cellular glucose uptake via the inhibition of Akt-mediated membrane translocation of Glut4 channels [59]. Ceramide also inhibits the cellular uptake of amino acids and fatty acids by downregulating the plasma membrane expression of their respective transmembrane transporters (sodium-coupled neutral amino acid transporter 2 and CD36, also known as fatty acid translocase) [60].

Ceramides are well known to initiate cell cycle arrest and apoptosis [61]. Ceramide-mediated inhibition of Akt provides a partial basis for its role in promoting cell cycle arrest, inhibition of cell growth, and apoptosis [19]. Physiologically, Akt activates anabolic cellular pathways including protein synthesis, promotes nutrient storage, and activates enzymes that promote cell growth and survival and inhibit autophagy [62]. Ceramide inhibits almost all of these processes, and in doing so, facilitates activation of caspase-9, an enzyme normally inhibited by Akt, which induces the apoptosome and ultimately leads to apoptosis [63]. Ceramide also promotes apoptosis through other signalling pathways such as initiating protein phosphatase 2A (PP2A)-mediated dephosphorylation and deactivation of the anti-apoptotic B-cell lymphoma 2 (BCL-2) protein and altering plasma membrane structures to trigger clustering of death receptors such as Fas/CD95 on the plasma membrane [64]. Finally, mitochondrial ceramides facilitate the release of mitochondrial intermembrane space pro-apoptotic proteins, an important step in the cell-death pathway [61]. Upregulation of mitochondrial ceramide production along with ceramide-mediated recruitment of BCL-2 family proteins Bax and Bcl-xL results in the formation of large stable pores in the outer mitochondrial membrane allowing the exodus of pro-apoptotic proteins [61]. Whilst these represent the best-characterised mechanisms of ceramide-induced apoptosis, in reality, there are a vast number of mechanisms through which ceramides trigger cell death [65].

The role of dihydroceramide in inducing cell growth arrest and apoptosis is controversial [66]. A small number of studies suggested that the accumulation of dihydroceramide induced apoptosis in a number of cell types [67]. Many studies, however, have not been able to replicate such pro-apoptotic effects [68]. Evidence shows that the lack of the 4–5 double bond diminishes dihydroceramide’s ability to form mitochondrial membrane pores as ceramide does [69]. Some studies even report that desaturase inhibition makes cells more resistant to pro-apoptotic stimuli in vitro [70].

Ceramides have an important role in maintaining the structure and function of the epidermis [71]. Current knowledge of stratum corneum structure proposes a ‘brick and mortar’ in which the corneocyte ‘bricks’ are embedded in an adhesive ‘mortar’ of lipid bilayers comprising ceramides, sterols, cholesterol, and fatty acids [72]. This contributes to the formation of a hydrophobic lamellar network acting as an impermeable envelope preventing epidermal water loss [71]. Sphingomyelin and glycosphingolipids like glucosylceramide are also important to ceramide epidermal function as they are present in the stratum corneum as precursors [73]. Inhibition of ceramide synthesis results in delays to epidermal barrier repair following damage [74].

#### 2.2.3. Sphingomyelin

Sphingomyelin is an important signalling molecule and has key functions related to plasma membrane structure and trafficking, DNA integrity and transcription regulation, cell proliferation, adhesion, apoptosis, and neural tissue development [75]. Sphingolipid is found most abundantly in the plasma membrane, where it is critical to promoting stability and maintenance of the bilayer structure, owing to its relative inertness [76]. Sphingomyelin can also associate with cholesterol to create a so-called liquid-ordered phase, from which disparate lipid domains, known as lipid rafts, can form [76]. Sphingomyelin may also have a role in Golgi-plasma membrane protein trafficking and secretion as inhibition of SMS in HeLa and other animal cell lines leads to impaired trafficking and secretion of both membrane proteins and secreted proteins such as insulin [77]. The formation of lipid microdomains in the Golgi and the plasma membrane appears key to vesicular trafficking between the two [75].

Some limited evidence exists implicating sphingomyelin in the regulation of nuclear transcription as sphingomyelin was shown to upregulate EcoRII cytosine DNA-methyltransferase-mediated DNA methylation in vitro [78]. Sphingomyelin also seems to bind and stabilise DNA in hepatocytes throughout liver regeneration, suggesting a protective effect on DNA [79]. Sphingomyelin is essential for eukaryote viability, growth and survival, and inhibition of sphingomyelin synthesis obliterates cell growth and hastens cell death in human and animal cell lines [80]. Plasma membrane sphingomyelin was shown to be essential to create optimal conditions for cell adhesion [81]. Whilst sphingomyelin does not seem to be directly involved in modulating cellular apoptosis, modulation of the balance of intracellular ceramide levels by SMS and SMase enzyme activity indirectly influences cellular susceptibility to apoptotic signals [75]. Finally, like glycosphingolipids, sphingomyelin is also found in the myelin sheathes of the peripheral (PNS) and central (CNS) nervous systems, though they are nowhere near as abundant as glycosphingolipids [82].

#### 2.2.4. Glycosphingolipids

In addition to acting as precursors for skin ceramides, glycosphingolipids in general occupy a vast range of functions that vary according to their individual chemical structures and properties. Many different glycosphingolipids are critical to membrane organisation and are found in lipid rafts, predominantly in the external membrane leaflet [83]. The length of fatty acid chains present in glycosphingolipids means that these external membrane constituents can extend far into the inner leaflet, a feature that may facilitate the linking of extracellular stimuli with intracellular signalling pathways [84].

Glucosylceramide in particular is implicated in intracellular membrane transport, cell differentiation, lymphocyte function, anticoagulation, cell survival and drug resistance in cancer therapy, and modulation of insulin signalling amongst many others [5]. Evidence suggests that membrane-localised glucosylceramide is important in endosomal trafficking and that depletion of glucosylceramide disrupts endosomal targeting [85]. Studies in myeloid cell lines report upregulated generation of glucosylceramide during cell differentiation, potentially suggesting a role for glucosylceramide in cell differentiation [86]. Glucosylceramide also appears to have a role in negatively regulating immune activation, specifically natural killer T (NKT) cells and subsequent immune-mediated inflammation [24]. Both in vitro and in vivo studies demonstrate that β-glucosylceramide presented by the CD1d major histocompatibility complex class I-like ligand inhibits NKT activation and this seems to be the case in humans as well [87]. Administration of glucosylceramide ameliorates inflammatory damage in animal models of hepatitis, colitis, and diabetes [88]. Glucosylceramide may also have a role in preventing thromboembolism, acting as an anticoagulant cofactor that enhances sensitivity to activated proteins C and S [89]. Cancerous cells use glucosylceramide to become resistant to chemotherapy agents and promote cell survival [90]. Many chemotherapy agents work by increasing the cellular production of the pro-apoptotic ceramide [91]. Cancer cells can develop resistance to these cytotoxic agents by upregulating the metabolism of ceramide into glucosylceramide [90]. Studies show that inhibiting UGlu-CG ameliorates the drug-resistant phenotype [92]. Finally, glucosylceramide is an independent antagonist of cellular insulin signalling, like ceramide, and seems to have a role in the development of insulin resistance, via incompletely understood mechanisms that may implicate downstream glycosphingolipids such as monosialodihexosylganglioside (GM3) [93].

Galactosylceramide is a key component of the myelin sheath in both the CNS and PNS, like sphingomyelin, and modulates lymphocyte function and systemic inflammatory signalling [24]. Animal studies have demonstrated the importance of galactosylceramide in CNS myelin formation, axo-glial interactions at the nodes of Ranvier, and axo-glial adhesion [94]. Current theories propose that trans-carbohydrate-carbohydrate interactions between galactosylceramide and 3-sulfo-galactosylceramide, a sulphated derivative, are key to the formation of a functional, multilayered myelin sheath structure, and may also facilitate signalling between adjacent oligodendrocytes [95]. Animal and in vitro studies report that 3-sulfo-galactosylceramide inhibits oligodendrocyte terminal differentiation and maturation, whilst inhibition of the 3-sulfo-galactosylceramide synthetic enzyme rescues the phenotype [95]. Contrary to glucosylceramide, galactosylceramide, specifically α-galactosylceramide, has been shown to activate NKT cells when presented with CD1d and consequently induce cytokine production and activation of the innate and adaptive immune systems [96]. α-Galactosylceramide’s immune-stimulatory signalling may have a therapeutic use in the promotion of immunotolerance and prevention of autoimmune disease and has already demonstrated efficacy in animal models of type 1 diabetes mellitus (T1DM) [96].

#### 2.2.5. Ceramide-1-Phosphate

Phosphorylated sphingolipids are important signalling molecules, and C1P signalling is implicated in cell survival, growth, proliferation, and migration as well as regulation of inflammation [5]. Contrary to the pro-apoptotic ceramide, C1P prevents cell apoptosis and promotes survival through a number of mechanisms [97]. C1P was shown to directly downregulate Ac-SMase activity in macrophages, thereby diminishing cellular ceramide production [98]. C1P also blocks caspase activation, prevents DNA fragmentation, and upregulates pro-survival signalling via the Akt/PI3K pathway and Bcl-XL, an antiapoptotic member of the BCL family [98]. C1P was initially shown to promote cell growth by upregulating DNA binding to nuclear factor kappa beta (NF-κB) and inducing DNA synthesis [99]. Subsequent studies in macrophages show that C1P stimulates cellular proliferation through extra-cellular signal-regulated kinase 1/2, c-Jun N-terminal kinase (JNK), and PKCα activation [100]. Extracellular C1P also triggers macrophage migration as well as proliferation, seemingly through an as-of-yet undiscovered ‘C1P receptor’ [101].

C1P modulates systemic inflammatory physiology in two critical ways [97]. Firstly, C1P is a direct and specific activator of cytosolic phospholipase A2, which regulates the generation of arachidonic acid, and its metabolism to all downstream eicosanoids [102]. Given the importance of eicosanoids to the pathophysiology of chronic inflammatory and cardiovascular conditions, C1P’s role in their activation makes it an attractive therapeutic target against these disease processes. More recent research has shown that C1P is a potent and direct inhibitor of the tumour necrosis factor-α (TNFα)-converting enzyme (TACE), an enzyme critical to the cleavage and liberation of TNFα from pro-TNFα [103], further adding to its inflammation-regulating potential.

#### 2.2.6. Sphingosine-1-Phosphate

S1P has been implicated in cell survival, autophagy, inflammation, immune cell migration, and various aspects of vascular biology [5]. S1P has been shown to promote cell survival and protect against apoptosis through PI3K- and PKC- mediated downregulation of the proapoptotic Bim protein and upregulation of the antiapoptotic-induced myeloid leukaemia cell differentiation protein-1 which in turn inhibits caspase-3 cleavage [104]. S1P is also critical to the merging of the lysosome and autophagosome, thus, making S1P an important modulator of cellular autophagy [105].

S1P has a crucial role in immune cell trafficking and immune response modulation [106]. S1P is a signalling molecule that can bind any of five g-coupled protein receptors (S1PR1-5) [107]. These receptors initiate signalling pathways involved in the modulation if innate and adaptive immunity and maintenance of endothelial barrier integrity [107]. Whilst most cell types will express at least one S1PR, these receptors appear to be differentially expressed in immune and vascular endothelial cells [108]. S1PR1 activation is integral for cell migration, the most well-characterized example being the egress of T and B lymphocytes from lymphoid tissues into the lymphatic and vascular circulations [106]. In order to facilitate the trafficking of lymphocytes out of their respective sites of maturation within lymphoid organs, an S1P gradient is generated: S1P levels are high in the blood and lymph but low in lymphoid tissues [109]. This is achieved via increased S1P production by erythrocytes and vascular and lymphatic endothelial cells combined with an increased activity of S1P degrading enzymes in the lymphoid organs [110]. Inhibition of SPL disrupts the S1P gradient by oversaturating tissues with S1P leading to a failure of peripheral lymphocyte migration, thereby exhibiting the importance of S1P to immune cell trafficking [111].

Two of the largest sources of extracellular S1P in the body are platelets and erythrocytes, both of which routinely release S1P into the blood [40]. This plasma S1P, bound to apolipoprotein M (ApoM)-anchored high-density lipoprotein cholesterol (HDL) (65%) or albumin (35%), has a number of presumed physiological functions in the blood and the vasculature [112]. A significant body of evidence suggests that S1P signalling can be pro-angiogenic, especially when mediated through the S1PR1 [113]. Recently, Balaji Ragunathrao et al. demonstrated that S1PR1 mediates the phosphorylation of vascular endothelial growth factor receptor 2 (VEGFR2) via activation of non-receptor tyrosine kinase c-Abl1 resulting in the persistence of VEGFR2 on the endothelial cell plasma membrane [114]. Indeed S1PR1 antagonism with fingolimod inhibits angiogenesis and tumour vascularisation [115]. Other studies, however, report that, in mice, S1PR1 knockout inhibits VEGFR2 phosphorylation and downregulates the sprouting of new vessels [113]. Sarkisyan et al. suggest a more complex model in which high and low extremes of S1PR1 activation stunt angiogenesis whilst mid-range S1PR1 signalling is pro-angiogenic [116].

S1P is also crucial to the maintenance of endothelial barrier integrity and fluctuations in S1P metabolism may modulate vascular permeability [117]. S1P induces the assembly of intercellular adherens and tight junctions [118]. S1P also acts via S1PR1 to suppress matrix metalloproteinases (MMPs) thus preventing endothelial glycocalyx shedding and remodelling as well as stimulating glycocalyx synthesis [119]. S1P-deficient mice suffering anaphylaxis or histamine administration experience increased vascular leakage and mortality compared to controls, a phenotype that is ameliorated by the administration of erythrocytes, one of the main sources of plasma S1P [120]. Furthermore, knockout of both SphK and S1PR1 leads to vascular leak, haemorrhage, and early embryonic death in mice [121,122]. Whilst S1P is generally considered to promote endothelial integrity, evidence also shows that at higher concentrations and S1PR2 signalling pathways, S1P can also instigate increased endothelial permeability [117].

Therefore, S1P has an important role in directly and indirectly modulating vascular inflammation [123]. Maintenance of endothelial barrier integrity indirectly suppresses vascular inflammation by reducing the accumulation of pro-inflammatory plasma proteins such as leukotrienes and complement in the intima [123]. On the other hand, the ApoM-S1P moiety of HDL-anchored S1P appears to be responsible for some of the direct anti-inflammatory effects of HDL, including suppressing inflammatory responses in endothelial cells triggered by cytokines [124]. These suppressive effects of S1P-ApoM-HDL are absent with ApoM deficient HDL and levels of S1P-ApoM-HDL have been shown to be much lower in inflammatory cardiovascular diseases including atherosclerosis and T2DM [125].

## 3. Sphingolipids and Vascular Disease

There is a strong evidence base for both protective and deleterious roles of different sphingolipids in vascular diseases such as atherosclerosis, stroke, and aneurysmal disease (Table 1).

### 3.1. Sphingolipids and Atherosclerosis

Ischaemic heart disease (IHD), characterized by the development of atherosclerotic plaques in the coronary arteries, is the leading cause of cardiovascular morbidity and mortality worldwide [1]. Atherosclerotic plaques build up over many years and, if stable, give rise to chronic coronary occlusion that typically manifests with chest pain and reduced exercise tolerance, both of which negatively impact the quality of life. When unstable, atherosclerotic plaques may rupture and/or embolise to cause acute total coronary occlusion, precipitating a myocardial infarction (MI). Atherosclerosis is a complex phenomenon that combines pathophysiological processes involved in dyslipidaemia, inflammation, insulin resistance, oxidative stress, and hypertension to culminate in the formation of atheromatous plaques in the coronary arteries [126]. Such plaques will grow until they eventually cause critical coronary artery occlusion either via progressive luminal stenosis or acute events such as plaque rupture and thrombosis or embolism [126]. Given sphingolipids’ numerous roles pertaining to inflammation and immune cell modulation, cell migration, and endothelial barrier function, it is unsurprising that sphingolipids are implicated in the pathophysiology of atherosclerosis.

At an observational level, plasma levels of most sphingomyelin and ceramide species are raised in patients with coronary artery disease [127]. Multiple studies have shown that plasma ceramides, dihydroceramides, sphingomyelins, and sphingosine predict IHD incidence and major cardiovascular events (MACE), including cardiovascular death, independently of other IHD risk factors [128]. Elevated levels of ceramide, dihydroceramide, glucosylceramide, lactosylceramide, sphingomyelin, and S1P are a known characteristic of advanced atherosclerotic plaques (>70% stenosis) and are more likely to be associated with the presence of angina in known coronary disease [129]. Ceramides appear to be key mediators of the deleterious signalling implicated in IHD. Ceramides C16 and C18, more so than other members of the species, are predictive of increased risks of coronary artery disease, thrombosis, cardiovascular death, and all-cause mortality [130]. Notably, ceramide-deficient mouse models display reduced atherosclerotic lesion burden, macrophage infiltration, lipid deposition, and inflammatory factor activation [131]. Crucially, mechanistic studies have proposed causal atherogenic roles for ceramides, as well as other sphingolipids, through a combination of inflammation, vascular smooth muscle cell dysfunction, vascular redox dysregulation, dyslipidaemia, and endothelial dysfunction as detailed below [132].

Inflammation and a dysregulated redox state are hallmarks of atheroma formation [126]. Significant positive correlations exist between plaque sphingolipids and key atherogenic inflammatory markers such as monocyte chemoattractant protein-1 (MCP-1) and interleukin-6 (IL-6) [glucosylceramide, lactosylceramide, ceramide, dihydroceramide, sphingomyelin]; and TNF-α and regulated on activation, normal T cell expressed and secreted (RANTES) [S1P] [129]. Plaque glycosphingolipid levels also correlate with plaque CD68 macrophage levels [129]. In vitro experiments show that glucosylceramide in particular is a potent stimulator of human coronary artery smooth muscle cell (HCASMC) macrophage inflammatory protein-1β, TNF-α, MCP-1, and RANTES secretion and of HCASMC apoptosis [129].

Reactive oxygen species (ROS) and ceramide species also appear to mutually upregulate each other in atherosclerosis [132]. Though exact mechanisms are unclear, it is thought that ceramide-enriched cell membrane domains may recruit and upregulate the activity of enzymes such as nicotinamide adenine dinucleotide phosphate (NADPH) oxidase (NOX) and endothelial nitric oxide synthase (eNOS) to upregulate ROS production [133]. Sphingolipids can also upregulate cellular ROS production through mitochondrial ceramide accumulation, which in turn, permeabilises the outer mitochondrial membrane facilitating cytochrome-c release, electron transport chain disruption, and subsequent ROS production [134]. Simultaneously, ROS may also upregulate ceramide synthesis via activation of Ac-SMases and N-SMases, which subsequently convert sphingomyelin to ceramide [135]. These pathways are corroborated by Ji et al. in which TNFα administered to human umbilical vein endothelial cells (HUVECs) triggers significant ceramide-mediated ROS production, which can be ablated with co-administration of an Ac-SMase inhibitor (amitriptyline) [136]. Ceramide-mediated ROS production may have exogenous triggers as well; Scheitzer et al. demonstrate that endothelial exposure to cigarette smoke also activated N-SMase-mediated ceramide production, leading to ROS accumulation and subsequent p38 MAPK/JNK/Rho kinase-mediated endothelial barrier disruption [137].

Through upregulating oxidative stress, ceramides inherently induce endothelial dysfunction through ROS-mediated uncoupling of eNOS leading to reduced nitric oxide (NO) bioavailability, increased ROS production and resultant vasoconstriction, and increased vascular permeability [57]. Ceramide has also been suggested to activate PP2A, which in turn, directly attenuates eNOS activation through both direct eNOS dephosphorylation and decreased phosphorylation of the Akt necessary for eNOS activation [138]. Ceramide-induced reduced NO bioavailability leads to loss of the vasorelaxant, anti-inflammatory, antithrombotic, and antihypertensive benefits of NO on the vascular endothelium [139]. Accordingly, endothelial cell-specific SPT knockout mice exhibit absent ceramide synthesis, increased eNOS activity and NO production, and decreased vasoconstriction and blood pressure [139]. In sufficient levels, ceramide-induced mitochondrial outer membrane permeability can also trigger caspase activation and lead to endothelial cell apoptosis, further debilitating endothelial function [140]. Whilst some evidence shows, counterintuitively, that exogenous ceramide treatment can upregulate eNOS mRNA and protein expression levels in HUVECs, the phenotypic reduction in NO bioavailability remains consistent [141]. It may be that this represents an attempt at compensation to match ceramide-mediated ROS production, or alternatively, that even if ceramides do induce a direct net positive effect on eNOS production in some endothelial cells, their overwhelming upregulation of ROS production may outweigh any eNOS-mediated upregulation of NO production and oxidise the NO, thereby diminishing its bioavailability [141].

Lipoprotein aggregation and accumulation in the intima media is a key feature of atherosclerosis development and appears to be directly and indirectly mediated by ceramide signalling. SMase-mediated conversion of polar, hydrophilic, sphingomyelin molecules present in low-density lipoprotein cholesterol (LDL) particles to ceramide leads to the formation of hydrophobic LDL surface domains, thus promoting aggregation with other LDL particles [142]. Increased LDL ceramide content may also lead to conformational changes in the critical apolipoprotein B100 (ApoB100) protein, resulting in exposure of specific ApoB100 domains prone to LDL particle aggregation [143]. Inhibition of de novo ceramide synthesis with myriocin (an SPT inhibitor) produces LDL particles less prone to aggregation and with lower ceramide and SMase concentrations thus confirming a key role for sphingolipids in promoting LDL aggregation [144]. Ceramides are also implicated in the transcytosis of oxidised LDL particles (ox-LDL) into the intima. Endothelial cell ceramide production stabilises cell membrane lipid rafts containing the Lox-1, Cavin-1, and Caveolin-1 proteins chiefly responsible for ox-LDL internalisation [145]. Again, Li et al. show that SPT inhibition reduces expression of Lox-1, Cavin-1, and Caveolin-1; and reduces endothelial ox-LDL transcytosis and retention in both HUVECs and mice models [145]. Contrary to the deleterious effects of SMase sphingomyelin ameliorates dyslipidaemia by downregulating intestinal cholesterol absorption [146].

Finally, ceramides are known to modulate immune signalling to attract peripheral blood monocytes to the vascular endothelium and encourage cell-endothelial adhesion and differentiation into macrophages [132]. Treatment of monocytes with ceramide precursor palmitate, upregulates expression of CD11b (a key adhesion, migration and transmigration molecule) and CD36 (a scavenger receptor with an affinity for LDL), along with a correlated increase in cellular ceramide 16:0 levels [147]. Such observations are absent following treatment with other fatty acids, suggesting the implication of the ceramide de-novo synthesis pathway in some capacity [147]. As a result, monocytes were observed to exhibit increased vascular adhesion and ox-LDL uptake resulting in foam cell formation [147]. Furthermore, macrophages may secrete SMase capable of hydrolysing LDL-associated sphingomyelin to ceramide in order to promote LDL aggregation as discussed earlier, leaving LDL aggregates vulnerable to uptake by macrophages and foam cell formation [132]. Clearly, immune and endothelial cell ceramide signalling are implicated in immune cell recruitment and foam-cell formation.

Contrastingly, S1P appears to have both pro- and anti-atherogenic roles in atherogenesis. S1P’s lymphocyte trafficking functions increase the number of peripheral circulating lymphocytes [106]. As a result, S1P upregulates lymphocyte activation, adhesion molecule expression, and cell migration to the intima to contribute towards plaque growth and the development of atherosclerosis [148]. S1P may also instigate endothelial dysfunction through S1PR2 signalling [149]. A number of studies have shown that supraphysiological concentrations of S1P can induce vasoconstriction and increased vascular permeability [150]. In vitro experiments in human coronary artery endothelial cells and aortic endothelial cells, S1P administration reduces NO levels and eNOS activity as well as Akt and PI3K phosphorylation, whilst upregulating expression of cell adhesion molecules (VCAM-1 and ICAM-1) and levels of peripheral mononucleocyte adhesion [151]. Antagonism and knockout of the S1PR2 abrogate these effects, however, suggesting a role for S1PR2 mediated inhibition of Akt/PI3K signalling pathways to achieve S1P’s atherogenic effects [151]. That endothelial cell S1PR2 expression alone is upregulated in a hyperglycaemic environment whilst other S1PR expression is reduced may suggest that S1P is a key signalling intermediate linking diabetes and metabolic dysfunction with endothelial dysfunction [151].

On the other hand, S1P is bound to HDL cholesterol in the circulation and when bound to HDL, exists in a complex with ApoM; through this collaboration, has been shown to ameliorate pro-inflammatory endothelial cell signalling [124]. HDL-ApoM-S1P triggers the formation of a cell surface S1PR1-B-arrestin complex, which then inhibits TNFα-induced NF-κB signalling, reduces expression of endothelial cell adhesion molecules and subsequent monocyte adhesion, and preserves endothelial barrier integrity [124]. S1P may improve endothelial barrier function through a number of purported pathways including triggering assembly of vascular endothelial cadherin and catenins, both components of endothelial adherens junctions; stabilisation of existing cell-cell junctions; inducing rho-kinase-mediated cell spreading; and increasing endothelial cell motility [152]. S1P also promotes endothelial cell proliferation and survival and may have a role in enhancing stabilisation and repair of endothelial barrier disruptions through activation of AMP-activated protein kinase (AMPK) signalling [153].

Early in vitro studies suggest C1P may protect vascular wall integrity by promoting neointima formation and endothelial repair, though further confirmation and characterisation of this pathway are needed [154].

Glycosphingolipids have been shown to promote atherogenesis. Glycosphingolipids do not exist unbound in the plasma but rather are associated with circulating lipoproteins, chiefly LDL-cholesterol [155]. Glucosylceramide and lactosylceramide accumulate in the intima of atherosclerotic plaques and have been shown to exist there at levels higher than any other sphingolipid [129]. As mentioned earlier, Edsfeldt et al. demonstrate that glucosylceramide is the greatest inducer of pro-inflammatory cytokines in HCASMCs and, along with lactosylceramide and ceramide, induces HCASMC apoptosis [129]. Indeed, inhibition of glucosylceramide synthesis in mice reduced inflammatory gene expression and atherosclerotic plaque formation [156]. Lactosylceramide also exhibits pro-inflammatory and proatherogenic effects through upregulating endothelial cell expression of NF-κB and ICAM-1 [157].

Glycosphingolipids may also provide some protective benefits in atherosclerosis, however. Glucosylceramide has a known purported anticoagulant effect as discussed earlier [158]. Lactosylceramide has been shown to promote the proliferation of human vascular smooth muscle cells, a well-known protective mechanism employed by the human body in atherosclerotic disease to promote the formation of protective caps over atherosclerotic plaques to prevent rupture [159]. It is, therefore, possible that the upregulation of these glycosphingolipids in atherosclerotic plaques serves to reduce the risk of an unstable plaque or plaque rupture developing [91]. This is further supported by data showing that individuals with a history of MI had lower glucosylceramide levels than those with no history of MI and that lower glycosphingolipid levels were seen in intimal tissue affected by atherosclerosis compared to unaffected intima within the same patient cohort [159]. Interestingly, LDL exerts an inhibitory effect on the synthesis of the atheroprotective lactosylceramide, demonstrating the presence of lipoprotein-mediated regulation of sphingolipid metabolism [160].

### 3.2. Sphingolipids and Aneurysmal Disease

An aneurysm describes a localised dilation of a vessel, secondary to chronic weakening of the vessel wall and subsequent inability to withstand the blood pressure, which may result in rupture and catastrophic haemorrhage over time. Risk factors for the development of aneurysmal disease include increasing age, cigarette smoking, male sex, family history, inherited connective tissue disorders, hypertension, atherosclerosis, infection, inflammation, oxidative stress, and obesity [161]. The importance of obesity in aneurysm pathophysiology is increasingly being appreciated as it has become clear that adipose tissue is an endocrine organ secreting bioactive molecules rather than simply an energy store. Increased perivascular adiposity not only creates a pro-inflammatory local environment for the vessel but also predisposes to dysregulation of sphingolipid levels around the aorta typically resulting in increased levels of ceramide and sphingomyelin and reduced S1P levels [162].

Ceramide and sphingomyelin promote aneurysm development through inflammation, apoptosis, and atherogenesis. Both ceramides and increased adiposity predispose to increased numbers of inflammatory cells and cytokines [161,162]. Increased numbers of macrophages, in particular, are associated with increased secretion of inflammatory cytokines including TNFα, IL-6, and interleukin-1β (IL-1β), which in turn activate MMPs and promote ceramide synthesis [163]. Inducing apoptosis in a multitude of cell types is part of ceramide’s physiological function. Ceramide-induced vascular-smooth-muscle cell death specifically can predispose to aneurysm formation as loss of the surrounding smooth muscle layer further weakens the arterial wall [164]. Finally, as discussed previously, ceramides, glycosylceramides, and sphingomyelin are all implicated in atherogenesis, the presence of which itself predisposes vessels to aneurysmal progression [165].

C1P may also have a role in aneurysm pathogenesis despite displaying both anti- and pro-inflammatory activity. On one hand, C1P has been shown to inhibit cigarette-smoke-induced inflammation, specifically downregulating the expression of TNFα, 1L-1β, and IL-6 [166]. This is significant in the context of cigarette smoking being one of the largest risk factors for aneurysmal disease. However, despite this, C1P also activates MMP2 and MMP9, two of the key MMPs involved in aortic matrix destruction [167]. C1P also activates inflammatory cytokines IL-6 and TNFα and stimulates phospholipase A2 in order to promote prostaglandin biosynthesis [161].

S1P, contrastingly, is thought to be protective against aneurysmal disease as an aneurysmal disease is associated with lower plasma S1P concentrations [168]. This association is further supported by a trial of fenofibrate in mice that increased S1P concentrations and subsequently reduced aortic aneurysm progression [169]. S1P mediates its protective effects through signalling via the S1P receptor 1 or 2, triggering downregulation of endothelial cell adhesion molecule and upregulation of cyclooxygenase 2 (COX2) expression, respectively [170]. Both pathways result in reduced inflammation. S1P is capable of pro-inflammatory signalling via S1PR3, however, which stimulates COX2-mediated production of the pro-inflammatory prostaglandin E2 [171]. Coincidentally, analysis of human aneurysmal aortic tissue shows that in abdominal aortic aneurysms, S1PR3 levels are upregulated whilst S1PR2 levels are downregulated thus providing a pathological mechanism by which S1P contributes to the inflammatory environment needed for aneurysm formation [171].

### 3.3. Sphingolipids and Stroke

A stroke is defined as an acute neurological deficit lasting more than 24 h and secondary to a cerebrovascular insult [172]. Strokes are typically categorised as ischaemic (approximately 87% of cases) or haemorrhagic (13% of cases) [172]. Multiple animal and human observational studies report that levels of ceramides are significantly increased in the acute ischaemic or haemorrhagic brain and in the reperfusion period following the stroke [173,174,175]. Kang et al. show that hypoxia upregulates SPT activity leading to increased ceramide synthesis and subsequent neuronal apoptosis [176]. Ceramide levels accumulate specifically in the ischaemic core and peri-ischaemic regions [174]. In particular, elevated ceramides post-stroke tend to be long-chain ceramides [173]. This pattern is corroborated in rat models of middle cerebral artery occlusion [177]. Elevated ceramides link to increased rates of haemorrhagic stroke as they induce cerebral vasoconstriction and leukocyte adhesion predisposing to small herald haemorrhages, which then risk precipitating a haemorrhagic stroke [178]. In patients with ischaemic stroke, sphingolipid tissue trends including increased long-chain ceramide and decreased S1P levels are mirrored in the plasma also [179].

Evidence largely points towards upregulation of the de novo ceramide synthesis, possibly triggered by increased levels of ROS in the wake of the ischaemia, as the primary source of increased ceramide levels [175]. Ablation of the elevated ceramide levels by myriocin, an SPT inhibitor, along with evidence of increased activation of ceramide synthases following ischaemia supports the theory that the de novo pathway is directly responsible [177]. Current theories suggest that the accumulation of ceramide in the ischaemic brain leads to ROS generation, tissue damage, and apoptosis resulting in larger areas of ischaemia and poorer functional outcomes. Sphingolipids, as well as being implicated in the core ischaemic pathology, contribute to a number of clinical phenotypes often considered to be risk-factors for cerebrovascular ischaemia: hypertension, obesity, diabetes mellitus, and atrial fibrillation. Human and rat studies show that increased ceramide levels are associated with increased systolic blood pressure for example [180].

Conversely, modulation of glycosphingolipid metabolism may be protective against cellular damage in ischaemic stroke. Ramirez et al. demonstrate that in rat brain tissue subjected to hypoxic ischaemia, levels of complex gangliosides (a subvariant of glycosphingolipid) diminish, whilst simple gangliosides accumulate [181]. It has been postulated that in acute infarction, catabolism of complex to simple gangliosides occurs, contributing to enhanced tissue damage and diminished neurological recovery [182]. Subsequent animal studies support this theory as prevention of complex ganglioside metabolism with chloroquine; stimulation of complex ganglioside synthesis with L-threo-1-phenyl-2-decanoylamino-3-morpholino-1-propanol (L-PDMP); and even supplementation of rodent stroke models with monosialotetrahexosylganglioside (GM1), a complex ganglioside, have all been shown to prevent the metabolic shift from complex to simple gangliosides, resulting in reduced inflammation, oxidative stress, and cell death at the infarct site and improved neurological recovery [183,184,185]. Evidence, therefore, supports the protective role of complex gangliosides in ischaemic stroke; the next step involves identifying how to harness this sphingolipid subgroup as a viable therapeutic intervention.

Sphingolipids may also serve as powerful prognostic tools in cerebral ischaemia. Katajamäki et al. report their ceramide- and phospholipid-based cardiovascular risk (CERT2) score predicts MACE and successfully predicts stroke mortality in an elderly human population [186]. Human observational studies report that increased tissue and plasma ceramide levels are associated with both increased severity of ischaemia and poorer functional neurological outcomes [179]. Multivariate analysis of humans with acute ischaemic stroke undergoing endovascular thrombectomy demonstrates that plasma long-chain ceramide levels could be applied clinically to predict poor long-term functional outcomes [187].

## 4. Sphingolipids and Myocardial Disease

Dysregulated sphingolipid metabolism has also been implicated in disorders affecting the myocardium, chiefly cardiac failure and atrial fibrillation (Table 1).

### 4.1. Sphingolipids and Heart Failure

Heart failure (HF) is a clinical syndrome affecting approximately 1–2% of adults worldwide [188]. HF is characterised clinically by the inability of the heart to meet the circulatory demands of the body and is typically classified as preserved (>50%, HFpEF), reduced (<40%, HFrEF), or mildly-reduced (40–50%) left ventricular ejection fraction (LVEF) [189]. The causes of HF are varied and many and can be broadly categorised as due to ischaemia, myocardial disease, abnormal loading, and arrhythmia. Given the broad array of pathologies leading to HF, it is unreasonable to expect a single or set of sphingolipid signalling pathways to be solely responsible. Indeed, the roles of ceramides (discussed elsewhere in this review) in atherosclerosis and ischaemia, obesity, T2DM, lipotoxicity, and systemic inflammation will all be implicated directly or indirectly in the pathophysiology of HF [3]. Beyond this, the observed changes in sphingolipid metabolism in patients with HF may offer insights into potential new therapeutic strategies. Aside from the role of sphingolipids in atherosclerosis and arrhythmogenesis, which will be addressed elsewhere in this review, sphingolipids have also been shown to have a role in the pathophysiology of myocardial disease, subsequently leading to HF [190].

Patients with HF exhibit raised plasma ceramide and low plasma S1P levels [190]. In fact, a meta-analysis of two studies with more than 3000 patients either with or at risk of HF, reported ceramide 16:0 and phosphatidylcholine as the lipids most significantly associated with incident HF [191]. Plasma S1P and dihydrophingosine levels do not appear to be significantly associated with cardiac dysfunction [192]. Analysis of the cardiovascular health cohort (*n* = 4249) suggests that sphingomyelin 16:0 and ceramide 16:0 are both associated with an increased risk of HF, independent of ejection fraction, contrary to other sphingolipids [193]. In 2652 Framingham-study participants, increased plasma C16:C24 ceramide ratios were significantly associated with increased HF incidence and adverse cardiac remodelling. In chronic HFrEF, plasma long-chain ceramides, specifically C16, were predictive of increased MACE rates whilst very-long-chain ceramides (C22, C24) were predictive of reduced MACE rates [194]. In HFpEF, long-chain ceramides showed the same association; however, the inverse association with very-long-chain ceramides was not observed [195].

It is well known that de novo ceramide synthesis is upregulated in failing cardiac tissue and that ceramides accumulate in the myocardium as a result [196]. That myriocin and SPT knock-down both demonstrably reduce post-ischaemia remodelling and improve long-term indices of cardiac function such a fractional shortening and end-diastolic diameter suggests that the de novo ceramide synthesis at least plays some part in myocardial pathophysiology of HF [196]. Specifically in cardiomyocytes, ceramide accumulation in the inner mitochondrial membrane appears to be a key mechanism in disrupting the electron transport chain, instigating oxidative stress through the generation of ROS, and ultimately leading to induced cell death and impaired cardiac function [134,197]. Increased myocardial ceramide accumulation and ceramide-induced myocardial dysfunction are likely mutually causal processes as Chokshi et al. report that ventricular unloading in patients with advanced HF ameliorates myocardial ceramide accumulation [197]. Interestingly, HFpEF, despite classically being characterised by reduced apoptosis relative to HFrEF, nevertheless displays high myocardial ceramide levels. Dong et al. suggest that increased expression of anti-apoptotic proteins, including BCL-2, may offer a protective effect [198].

In addition to apoptosis, ceramides also induce myocardial disease through the regulation of myocardial inflammation and fibrosis [3]. Ceramides are involved in regulating both cytokine production and responses [199]. In murine models of ischaemic HF, overexpression of Ac-CDase and subsequent ceramide level reduction reduced myocardial immune cell infiltration and improved 28-day cardiac function [200]. S1P, on the other hand, is associated with reduced inflammation and physiological remodelling in mouse and human myocardium [201]. Meanwhile, evidence in other tissues suggests that ceramide modulates transcription factors involved in collagen production and may promote fibrosis, though these specific mechanisms have yet to be confirmed in the myocardium [3].

### 4.2. Sphingolipids and Atrial Fibrillation

Atrial fibrillation (AF) is the most prevalent cardiac arrhythmia and, due to its role in conferring increased risk of ischaemic stroke, it represents a significant health burden. AF typically requires a trigger and a substrate (vulnerable myocardial tissue that may sustain an abnormal rhythm) in order to manifest [202]. Risk factors for AF include oxidative stress, inflammation, and cardiac remodelling, specifically atrial fibrosis, all of which are processes inherently linked with sphingolipid signalling [202].

There is a paucity of evidence relating to the role of sphingolipids in the pathophysiology of AF with only a few observational studies. A large prospective cohort study of 4206 patients without cardiovascular disease at baseline reports that raised levels of sphingomyelin 16 are associated with increased incidence of AF over 8.7 years of follow-up [203]. Increased levels of ceramide 16:0 trended towards association with increased AF incidence but did not meet statistical significance [203]. Both associations were attenuated when adjusting for plasma NT-proBNP level, suggesting a mechanism of action resulting in increased atrial cardiomyocyte pressure loading may be implicated [203]. Contrastingly, increased levels of very-long-chain ceramides and sphingomyelins were associated with reduced AF incidence rates [203]. A more recent study also shows, in another prospective cohort of patients, that increased plasma S1P levels are observed at follow-up in those with AF compared to baseline and compared to those without AF [204].

Both tissue fibrosis and death are implicated in creating an arrhythmogenic substrate [202]. Furthermore, sphingolipids are implicated in both tissue fibrosis and cell death [202]. S1P seems to promote cardiac fibrosis in mice as demonstrated by overexpression of SphK and S1PR1 and 3 [205]. A similar molecular process drives fibrosis of other human tissues including the liver [206]. Ablation of SphK or administration of anti-S1P antibodies subsequently reduces tissue fibrosis [207]. Ceramides as modulators of inflammation, can also promote increased atrial fibrosis creating an arrhythmogenic substrate [202]. Indeed, inhibition of ceramide synthesis with myriocin, an SPT inhibitor, reduces myocardial inflammation and fibrosis [196]. Cardiomyocyte apoptosis likely also plays a role, as caspase-3 knock-down or inhibition in pigs and dogs, respectively, was shown to suppress AF [208,209]. Whilst ceramide is well known to promote caspase activation, S1P contrastingly is known to inhibit caspase activation and promote cell survival, thus suggesting added complexity to the role of S1P in promoting AF [104].

Finally, sphingolipids may promote arrhythmogenesis through dysregulation of cardiomyocyte membrane repolarisation [190]. S1P is known to regulate the inward rectifier potassium current through S1PR3 signalling and downstream muscarinic receptor activation in both human and animal cardiomyocytes [210]. S1P can, therefore, lead to the shortening of both the cardiomyocyte action potential and refractory period [210]. This, in turn, may promote genesis and potentiation of atrial fibrillation.

## 5. Sphingolipids and Metabolic Disease

A recent series of cross-sectional studies of 2063 middle-aged subjects from the United States (known as the MIDUS studies) reported positive associations between ceramides and the prevalence of obesity, dyslipidaemia, impaired glucose metabolism and metabolic syndrome [211]. There is a growing body of evidence implicating sphingolipids in the pathophysiology of the key components of metabolic disease, including obesity, inflammation, and insulin resistance.

Plasma long-chain ceramide and S1P levels are increased in both human and murine models of obesity [212]. Furthermore, increased plasma ceramide and S1P levels are positively correlated with body mass index (BMI), waist circumference, total cholesterol, LDL, and homeostatic model assessment of insulin resistance (HOMAIR) [213]. A number of mechanisms underpin dysregulated sphingolipid metabolism in states of obesity. Obesity is known to be characterised by increased circulating levels of saturated free fatty acids (FFAs); these increased circulating FFAs are associated with increased plasma and tissue ceramide levels and directly enhanced activation of the de novo ceramide biosynthesis pathway [214]. Saturated FFAs also activate the toll-like receptor 4 (TLR4), which further upregulates SMase-mediated ceramide biosynthesis through IκBβ signalling [215]. Obesity is also a state of inflammation and immune activation. Increased levels of TNFα in obesity can enhance the expression of SPT, Ac-SMase, and N-SMase, all of which contribute to enhanced ceramide synthesis [212]. This corroborates findings that long-chain ceramides are positively correlated with serum TNFα levels [216]. Furthermore, in vitro adipocyte experiments show that species of ceramide, S1P, and sphingosine upregulate the expression of pro-inflammatory (TNF-a, IL-6), pro-atherogenic (MCP-1), and pro-thrombotic (plasminogen activator inhibitor-1 (PAI-1)) biomarkers [212]. That both blockade and knock-out of both SPT and desaturase 1 (DES1) reduce body weight, insulin resistance and hepatic steatosis in multiple animal models further affirms the role of long-chain ceramides in the pathophysiology of metabolic dysfunction [217,218].

However, not all ceramides are equally harmful to metabolic disease. Heart, liver, and plasma levels of very-long-chain C24 ceramides are found to be reduced in obesity and diabetes, and their replacement is seemingly metabolically beneficial, increasing weight loss, insulin sensitivity, glucose tolerance, and FFA oxidation [219]. Indeed, genetically modified mice unable to produce long-chain ceramides (C22–24) are profoundly glucose intolerant despite normal pancreatic insulin secretion [220]. These mice displayed absent phosphorylation of both the insulin receptor and Akt, suggesting that long-chain ceramides may have a role in facilitating intracellular insulin receptor translocation, thereby protecting against the development of insulin resistance [220].

As discussed earlier, ceramides and glucosylceramides have a known role in the pathogenesis of insulin resistance thus rendering them complicit in the pathogenesis of T2DM. Plasma and skeletal muscle ceramide levels are significantly higher in diabetic than non-diabetic adults [216,221]. A recent genome-wide association study of over 5000 patients from multiple trials reports that enzyme-activity-enhancing genetic variations at the *sptlc3* gene locus, which encodes one of the SPT enzyme tetramers, contributed to significantly increased circulating ceramide levels and was associated with CVD and T2DM [222]. The rs680379 single nucleotide polymorphism was shown to downregulate SPT activity and reduce plasma sphingolipid levels as well as T2DM incidence [222].

Ceramides inhibit insulin signalling by diminishing Akt phosphorylation, whilst glucosylceramides act directly at the level of the insulin receptor to inhibit insulin signalling [93]. One particular complex glycosphingolipid, GM3, has been shown through cell and animal studies to be an especially potent inhibitor of insulin signalling [223]. Through dysregulation of insulin signalling, ceramides impair cellular uptake and storage of glucose and triglycerides [224]. Inhibition of de novo ceramide synthesis typically improves insulin sensitivity and has been shown to restore Akt phosphorylation in vitro [217,218]. Furthermore, adiponectin, a hormone well known for its insulin-sensitising properties, appears to exact its antidiabetic effects at least partially through induction of CDase-induced ceramide degradation [215]. Increased circulating adiponectin levels are associated with reduced tissue ceramide levels [225]. Likewise, glucosyltransferase enzyme inhibitors also increase insulin sensitivity and glucose tolerance in murine models of insulin resistance and obesity [226].

The immunomodulatory roles of sphingolipids may contribute to the pathogenesis of T1DM, typically characterized by autoimmune pancreatic β-islet cell destruction resulting in absolute insulin deficiency. Sphingolipids are known to be implicated in the pathophysiology of autoimmune diseases such as multiple sclerosis [5]. There is a general consensus that in T1DM, a combination of genetic predisposition and environmental triggers result in pathological crosstalk between macrophages and β-islets and subsequent release of pro-inflammatory cytokines including IL-1β, TNF-α, and IFN-γ that promote local inflammation, cell damage, immune cell recruitment, and through all of this, cell apoptosis [227]. Cell and animal studies have implicated sphingolipids in the pathogenesis of this autoimmune process. Rat insulin-secreting cells respond to IL-1β and IFN-γ signalling by upregulating CerS, N-SMase, and Ac-SMase, which in turn, leads to ceramide accumulation, mitochondrial damage, and cell death [228]. Pancreatic ceramide accumulation has also been reported in T2DM, and therefore, ceramides likely have some role in T2DM-associated β-islet cell destruction as well [229].

Contrastingly, Jörns et al. demonstrate that the use of fingolimod (an S1PR agonist) in T1DM rat models reduces pancreatic immune cell infiltration, cytokine expression, and β-islet cell destruction, resulting in improvements to both c-peptide secretion and maintenance of blood glucose levels [230]. The way in which adiponectin shifts the sphingolipid metabolic equilibrium towards increased S1P production results in increased S1PR activation, which upregulates Akt and AMPK signalling and subsequently enhances β-islet survival [215]. IL-1β-induced β-islet apoptosis is also abrogated by HDL-S1P signalling in mice and humans [231]. This suggests the S1P-S1PR signalling pathway may be a potential therapeutic target in T1DM prevention. S1P’s importance in maintaining metabolic health is further evidenced by the fact that SphK1 deletion in adipocytes results in adipocyte hypertrophy and insulin resistance [232]. S1P-S1PR signalling in metabolic disease is complex, however. It’s worth remembering that whilst enhanced S1P production usually implies reduced ceramide levels, in adipocytes, increased S1P signalling can be deleterious. S1PR2 activation upregulates the recruitment of pro-inflammatory macrophages and promotes pre-adipocyte differentiation and adipogenesis [233]. Further research is needed, therefore, to better characterise the role of S1P signalling through specific S1PRs in metabolic disorders.

## 6. Sphingolipids as a Therapeutic Target

Whilst there is copious evidence for the role of sphingolipids in CVD, there are currently no available licensed CVD treatments specifically targeted at sphingolipids. A key paucity in the field of sphingolipid therapeutics is the lack of in-human trials. Most evidence pertaining to potential drugs targeting sphingolipid signalling and metabolism stems from animal or cell studies.

### 6.1. Weight Loss and Exercise

Whilst obesity is inevitably implicated in the pathophysiology of sphingolipid metabolism, the nature of this association is complex, and the value of weight loss interventions remains uncertain.

A small number of heterogenous trials involving body mass loss and exercise have produced inconclusive results thus far. Dubé et al. compare the effects of diet-induced weight loss (*n* = 8) and aerobic exercise (*n* = 8), reporting that improvements in body weight and insulin sensitivity were present in both groups, but that reduction in plasma and muscular ceramide and sphingosine levels were only observed with aerobic exercise [234]. Similarly, a 12-week exercise plan in obese adults with and without diabetes reduced plasma levels of C14, C16, and C24 ceramides whilst significantly reducing body weight, adiposity, and peripheral insulin resistance [235]. On the other hand, 6 weeks of caloric restriction in non-diabetic, obese adults (*n* = 49) significantly improved plasma ceramide, dihydroceramide, and sphingomyelin levels [236]. One year of combined diet, exercise, and behavioural body-mass loss produced a similar effect in obese patients with steatohepatitis (*n* = 31) [237].

Studies in gastric bypass patients have also attempted to elucidate the benefits of ceramide profiles with more convincing results. A number of cohort studies mainly in Roux-en-Y gastric bypass (RYGB) patients demonstrate that RYGB is associated with significant reductions in plasma sphingomyelins and long- and very-long-chain ceramides (the unsaturated species in particular) [238,239,240,241,242,243]. These significant reductions in plasma sphingolipids are also shown to be independent of weight loss, implying that the mechanism through which gastric bypass improves sphingolipid profiles is more complex than simply weight loss [238]. Furthermore, the same studies also show strong correlations between decreases in sphingolipid species and HOMAIR and glycated haemoglobin, suggesting that alterations to plasma sphingolipid levels could be partially responsible for the metabolic benefits of RYGB beyond weight loss [238,239,241,242,243]. The exact mechanisms, however, remain to be characterised. One study measured brachial flow mediated dilatation pre- and post-operatively and found improved endothelial function, a trend that was inversely correlated with plasma ceramide levels [242]. Notably, a trial in 101 post-RYGB patients, reports that a 6-month exercise regime provided additional benefits to insulin sensitivity and intramuscular ceramide levels over health education and bariatric surgery and indeed was the only intervention to improve cardiorespiratory fitness, suggesting that exercise provides benefits beyond those mediated by surgery [244]. Mechanistic studies are the logical next step to try and identify the pathways through which RYGB-mediated sphingolipid reductions improve metabolic health.

### 6.2. Dietary Interventions

Dietary modification with the aim of improving sphingolipid profiles has been investigated in a number of different forms. Some studies have tested the value of dietary supplementation with compounds ranging from ginseng and oleic acid to phospholipids found in milk, whilst others have explored the efficacy of low-fat diets.

#### 6.2.1. Oleic Acid

Bikman et al. report that monounsaturated oleic acid inhibited hepatic and muscular DES1 expression in mice, thus reducing cellular ceramide accumulation and abating insulin resistance [218]. Hu et al. likewise report evidence that oleic acid specifically modulates DES1 expression both in myotubular cells and in mice [245]. A randomised, crossover trial in which healthy volunteers adopted three weeks of either a high palmitic acid (HPA) diet or a diet low in palmitic acid and high in oleic acid (HOA) produced similar results [246]. Following an HOA diet, female participants displayed improved insulin sensitivity along with reduced serum and muscle ceramide levels compared with an HPA diet [246]. It’s worth noting, however, that the same results were not seen in male participants and the study suffered generally from insufficient powering [246]. Nevertheless, there is reasonable evidence for a modest metabolic benefit from oleic acid, though larger studies with longer follow-up examining cardiovascular outcomes are needed to give any kind of insight into their utility in cardiovascular disease.

#### 6.2.2. Milk Polar Lipids

Milk contains a number of so-called polar lipids (MPLs) including milk sphingomyelin (MSM), which comprises 25% of all MPLs [247]. Whilst some studies have claimed that MSM can improve serum lipid profiles, the evidence overall is mixed [247]. The indication stems from the well-known capability of sphingomyelin to inhibit intestinal cholesterol absorption [248]. Rat studies have even suggested MSM to be the most effective variant in this respect [248]. In high-fat-diet mice, MSM reduces cholesterol absorption and, as a result, hyperlipidaemia and hepatic steatosis, whist increasing faecal cholesterol excretion [249,250,251]. Interestingly, there exists a body of data pointing towards a neutral, or even hyperlipidaemic, effect of MSM in various rodent models of obesity and metabolic dysfunction [247]. Furthermore, Le Barz et al., demonstrated that whilst some changes in sphingomyelin species abundance in intestinal chylomicrons and the serum were observed following MPL supplementation in post-menopausal women, ultimately there were no significant changes in total plasma sphingolipid levels [252].

One of the first clinical trials of purified MSM found that adult humans taking MSM supplements exhibited a modest increase in plasma HDL but with none of the hypolipidaemic benefits reported in rodents, possibly as human intestinal SMase is more effective than murine equivalents [253]. A more recent meta-analysis of 10 clinical trials of sphingomyelin supplementation in humans reported significant improvements to metabolic health characterised by reductions in serum insulin and the HOMAIR [254]. Weiland et al. also reported a significant reduction in waist circumference [255]. Effects on plasma lipid profiles, however, had mixed outcomes with significantly reduced total cholesterol and LDL and increased HDL reported in select low-dose subgroups, but no significant effects were seen at higher doses in the majority of studies, reflecting an overall limited effect on plasma lipids [254]. There is a paucity of literature on the anti-obesogenic effects of MPLs as a whole, though murine studies have shown reversal of high-fat-diet-mediated increases in total body mass, adipose mass, and adipocyte size in response to MPLs [256,257]. The mechanisms behind any potential anti-obesogenic effects are poorly understood. Two studies also measured the effect on blood pressure but found no significant changes [255,258]. Seemingly, the benefits of sphingomyelin supplementation seen in rodents have yet to be fully replicated in humans.

Similarly, studies examining the potential effects of broader diary consumption on plasma sphingolipid profiles and CVD outcomes have produced inconclusive results. A prospective study of 105 women reported that women who consumed higher levels of fresh dairy products had lower plasma ceramide and dihydroceramide concentration [259]. On the other hand, a randomised study in middle-aged men demonstrated that dairy products increased plasma levels of sphingomyelin, dihexosylceramide, and GM3 compared to soy alternatives [260]. Several recent meta-analyses report, in some cases strong, associations between increased consumption of total diary, cheese, and yoghurt and reduced incidence of IHD, stroke, and hypertension in adults [261,262,263,264,265,266]. Yet simultaneously, other meta-analyses report neutral associations between dairy consumption and CVD incidence and mortality [267,268,269]. The question of whether increased dairy consumption improves CVD incidence, morbidity, and mortality remains, as of yet, unanswered.

Overall, whilst there are some reasonable quality studies to support the benefits of MPLs and dietary sphingomyelin supplementation, the overall consensus is inconsistent and larger, controlled studies are needed to better understand the extent of the effects of MPLs and sphingomyelin on plasma lipids and metabolic health. Thus far there have also been no studies investigating MPL or sphingomyelin supplementation on long-term cardiovascular disease outcomes, and this represents a further unmet research need.

#### 6.2.3. Ginseng

There may be a role for the root of an ivy species from the Panax genus, known as ginseng, commonly used in herbal remedies and cooking in east Asia, in the treatment of vascular disease. A 2014 study in HUVECs reported that a ginseng extract known as ginsenoside compound K was able to inhibit S1P-mediated angiogenesis by downregulating SphK1 expression [270]. Ginsenoside compound K concomitantly increases cellular ceramide accumulation, specifically of the ceramide 24:0 species [270]. Contrastingly, in vivo and in vitro studies demonstrate that ginseng extracts can ameliorate tissue damage in models of myocardial and cerebral ischaemia and reperfusion through upregulation of intracellular sphingosine and S1P amongst other candidates, leading to reduced infarct sizes and reduced expression of proinflammatory mediators such as TNFα and IL-6 [271,272].

A double-blinded randomised control trial (RCT) also showed that Korean red ginseng reduced ceramide 16:0 levels in post-menopausal women with hypercholesterolaemia, though did not report any outcome data [273]. A couple of studies support a role in HF as well, as treatment of doxorubicin-induced mouse HF models with total saponin of black ginseng reduced serum B-type and atrial natriuretic peptides (BNP and ANP) and improved myocyte morphology and inflammatory cell infiltration on histology [274]. More recently, Tian et al. reported that notoginsenoside R1, a different active medicinal compound extracted from Panax ginseng, downregulated SPT activity through AMPK signalling and resulted in enhanced ejection fraction, systolic and diastolic function, and myocyte histological arrangement [275]. There appear to be a number of possible mechanistic explanations for the purported cardiovascular benefits of ginseng that implicate sphingolipids; however, a larger volume of robust studies is needed to better characterise these mechanisms [276].

#### 6.2.4. Low-Fat and Low-Carbohydrate Diet

Twelve weeks of a healthy Nordic diet low in fat reduced plasma C18:1 ceramides in 104 overweight patients with metabolic syndrome compared to 96 others on a regular fat diet [277]. Another low-fat diet rich in fruit and vegetables significantly reduced levels of circulating ceramide and glucosylceramide species along with waist circumference in adults at risk of metabolic syndrome [278]. Similar results, albeit of lesser magnitude, were seen in a parallel low-carbohydrate diet [278]. Notably in all diets, a paradoxical rise in the C16:0 ceramide species only was observed [278]. Whilst inevitably it is to be expected that a low-fat diet would improve plasma profiles of a dietary lipid, it is likely that the types and sources of dietary fats consumed are just as critical, if not more so, than the volume of fat consumed.

There is reasonable evidence for an association between increased saturated fatty acid (SFA) consumption and pathological plasma sphingolipid profiles. A large study of 2860 Singaporeans demonstrates that high dietary SFAs are associated with increased plasma d16:1 ceramides, hexosylceramides, sphingomyelin, and S1P, whilst high polyunsaturated fat intake was associated with low plasma levels of the same species [279]. Similarly, a number of other studies have shown that overweight subjects who were overfed a high SFA diet experienced increased insulin resistance, intrahepatic triglyceride deposition, and circulating plasma ceramide levels, specifically C14:0, 16:0, and 18:0 species, all of which were reversed with an unsaturated fat-enriched diet [280,281]. Indeed, animal and cell studies have shown that SFAs, Palmitate, in particular, directly upregulates de-novo ceramide synthesis and immune signalling activation, possibly via activation of the TLR4, and indirectly compromises insulin signalling to promote insulin resistance [282,283].

Data pertaining to unsaturated fatty acids (UFAs) are much more limited. Studies in rodent cells and tissues, interestingly, report that diets high in UFAs, traditionally considered deleterious in the context of CVD, seem to reduce intracellular ceramide and sphingomyelin accumulation and, in some studies, cardiomyocyte apoptosis [284,285,286]. Perhaps less surprising is that, despite reductions in tissue sphingolipid levels, high UFA diets still induced hepatic steatosis and insulin resistance in mice [286]. One mechanistic in vitro study demonstrated that UFAs induce hydrolysis of sphingomyelin back to ceramide though how this fits with results reporting reduced intracellular ceramide levels is unclear [287].

At present, human data on the role of different fatty acid subtypes are too sparse to draw any meaningful conclusions. Whilst in vitro, unsaturated fatty acids may have an adverse effect on intracellular sphingolipid accumulation, their role in sphingolipid-mediated CVD is likely far more complex given the robust data demonstrating their larger pathological impact on human metabolic health.

### 6.3. Pharmacological Treatments

**Figure 2:**
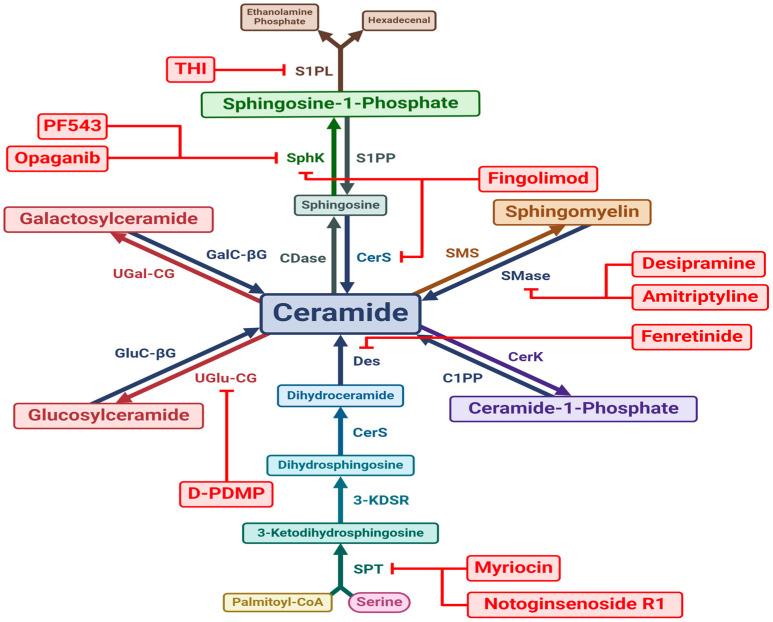
A figure displaying a schematic representation of the sphingolipid metabolic network along with the different classes of medication that directly target sphingolipid signalling and metabolism and the specific mediator(s) of sphingolipid signalling and metabolic pathways that they affect. SPT: Serine Palmitoyl Transferase, 3-KDSR: 3-Ketodihydrosphingosine Reductase, CerS: Ceramide Synthase, Des: Dihydroceramide Desaturase, SMS: Sphingomyelin Synthase, SMase: Sphingomyelinase, CDase: Ceramidase, SphK: Sphingosine Kinase, S1PP: Sphingosine-1-Phosphate Phosphatase, S1PL: Sphingosine-1-Phosphate Lyase, UGal-CG: UDP Galactose Ceramide Galactosyltransferase, GalC-βG: Galactosylceramide β-Galactosidase, UGlu-CG: UDP Glucose Ceramide Glucosyltransferase, GluC-βG: Glucosylceramide β-Glucosidase, CerK: Ceramide Kinase, C1PP: Ceramide-1-Phosphate Phosphatase, THI: Tetrahydroxybutylimidazole, S1PR: Sphingosine-1-Phosphate Receptor, D-PDMP: D-threo-1-phenyl-2-decanoylamino-3-morpholino-1-propanol. Different colours distinguish the unique products of individual enzymatic processes (for example arrows and enzymes that directly lead to ceramide generation are in dark blue etc.). Created with BioRender.com.

One of the key pharmacological challenges of targeting sphingolipid signalling and metabolism is that the physiological importance and ubiquity of sphingolipids increase the risk of off-target effects. Nevertheless, some limited success in pharmacologically targeting sphingolipid metabolism (Figure 2) has been reported, chiefly in animal trials.

#### 6.3.1. Fingolimod

To date, the most successful, and only licensed, example of direct pharmacological targeting of sphingolipid synthesis is the S1PR modulator fingolimod. Fingolimod is a sphingosine analogue and negatively regulates ceramide and S1P synthesis through inhibition of ceramide synthases, Ac-SMase, and SphK [288,289,290]. Fingolimod’s immunomodulatory effects have so far made it an effective therapy for reducing relapse rates in patients with relapsing-remitting multiple sclerosis [291]. Fingolimod can also act as an S1PR activator. Fingolimod is phosphorylated by SphK after which it binds and internalises S1PR1 on lymphocytes (acting as a functional antagonist), thus preventing lymphocyte migration into the systemic plasma and lymphatic circulations, thus justifying its value in multiple sclerosis [291].

In general, fingolimod tends to display cardioprotective effects, although some studies report deleterious cardiovascular adverse effects [292,293]. In addition to its peripheral immunomodulatory effects, fingolimod reduces atherosclerotic plaque size and composition, inhibiting the formation of a necrotic core and accumulation of CD3+ lymphocytes in LDL receptor (LDL-R) knock-out mice [294]. A study in MI-induced pigs reports fingolimod reduces infarct size, mass, percentage and transmurality and improves post-MI indices of recovery including myocardial salvage, LVEF, and contractile reserve [295]. In rat models of cardiac ischaemia and reperfusion, fingolimod reduces rates of ischaemia- and reperfusion-induced arrhythmia [296]. Fingolimod may also show benefits in the prevention of cardiac remodelling as it diminishes cardiac hypertrophy and restores normal cardiac structure and function in cardiac hypertrophy-induced mice [297]. Santos-Gallego et al. also report reduced cardiac remodelling and hypertrophy in pigs at 1 and 4 weeks post-MI [295]. However, multiple RCTs examining the efficacy of fingolimod in multiple sclerosis report serious adverse effects including hypertension, bradycardia, and first and second-degree atrioventricular conduction block requiring patients to be withdrawn from these studies [292,293]. Whilst fingolimod may demonstrate beneficial cardiovascular properties, its well-evidenced cardiovascular adverse effect profile is an important challenge to overcome if it is to be of clinical benefit to patients with cardiovascular disease.

#### 6.3.2. Statins

Multiple studies also demonstrate the efficacy of statins in lowering plasma sphingolipid levels in patients with metabolic disease. Ng et al. report rosuvastatin lowers a range of plasma sphingolipids including sphingosine, sphingomyelin, ceramide, and dihydroceramide [298]. Tarasov et al. similarly show that simvastatin lowers plasma ceramide levels by approximately 25% [299]. A trial in rabbits reports that 40 mg of atorvastatin daily not only reduces plasma and aortic ceramide and Ac-SMase levels but also reduces atherosclerotic plaque thickness and macrophage content and improves plaque stability [300]. On the contrary, ezetimibe does not reduce plasma ceramide levels and, in fact, provokes a mild elevation of two ceramide species [299]. The same study reported that 19 individuals with a protein convertase subtilisin/kexin type 9 (PCSK9) loss-of-function mutation demonstrated significantly diminished plasma ceramide levels, equivalent to the effect seen with statins, suggesting that PCSK9 inhibitors may also be a plausible therapeutic option in the future [299]. The comparatively larger effect size of PCSK9 inhibition compared to simvastatin implicates the LDL-R in mediating the clearance of plasma ceramide species.

#### 6.3.3. Glucagon-like Peptide-1 Receptor (GLP-1R) Agonists

Akawi et al. demonstrate a clinically significant reduction in plasma sphingomyelin, ceramide, and glucosylceramide levels in healthy, obese adults following a one-year course of daily liraglutide [301]. With no concurrent change in BMI compared to low-carbohydrate diet controls, this effect is seemingly independent of weight loss. Similarly, Denimal et al. report that 6 months of daily liraglutide in 86 T2DM patients reduces plasma levels of dihydroceramide and most ubiquitous ceramide species, but alongside a significant reduction in BMI compared to controls [302]. The same study also reports reduced liver fat content and improved indices of insulin resistance. Leonardi et al. support the therapeutic potential for GLP-1R agonists, demonstrating that Exendin-4 inhibits the palmitate-mediated activation of ceramide biosynthesis in human cardiac progenitor cells [303]. Reduced ceramide accumulation subsequently diminishes JNK activation and resultant cell apoptosis [303]. There may well be a role for GLP-1R agonists in cardiovascular and cardiometabolic disease; however, further studies are needed to elucidate whether this effect is independent of weight loss.

#### 6.3.4. Sodium Glucose Transporter 2 (SGLT-2) Inhibitors

SGLT-2 inhibitors are increasingly a mainstay of cardiovascular disease management having recently been licensed for cardiac failure in addition to T2DM. A recent study in diabetic rats reports that despite increased body weight and unaltered cholesterol profiles, diabetic rats on empagliflozin had lower cardiac levels of ceramide, sphingomyelin, and proinflammatory mRNA [304]. It is unclear whether these effects are direct or indirect and further mechanistic studies are needed.

#### 6.3.5. Amitriptyline

As well as inhibiting the transport of serotonin and noradrenaline, tri-cyclic antidepressant (TCA) amitriptyline also inhibits Ac-SMase and may have a role in treating cardiovascular disease [136]. Studies demonstrate amitriptyline’s inhibition of A-SMase is associated with reduced cell apoptosis [305,306]. These apoptotic pathways may be the same pathways implicated in ischaemia-reperfusion injury post-MI [306]. Studies in LDL-R knock-out mice show that amitriptyline attenuates aortic atherosclerosis and reduces hepatic steatosis, insulin resistance, and plasma glucose and lipid levels [307]. Unexpectedly, plasma ceramide and dihydroceramide levels were raised, despite inhibition of the ceramides salvage pathway, suggesting that amitriptyline may also have yet-uncharacterised effects on the de novo ceramide synthesis pathway [307].

Ji et al. report that amitriptyline ameliorates endothelial dysfunction in HUVECs by increasing endothelial cell proliferation and viability, diminishing expression of endothelial cell adhesion molecules (VCAM- 1, ICAM-1, MCP-1) and inhibiting monocyte adhesion [308]. Amitriptyline also increased endothelial eNOS phosphorylation and NO production of ROS thus enhancing endothelial NO bioavailability and consequently endothelial relaxation [308]. Contrastingly, Guan et al. report no change to ROS production in murine endothelial cells [309].

Amitriptyline also comes with a notable cardiovascular adverse effect profile, however, including atrioventricular conduction disorders and arrhythmia [136]. In vitro studies have suggested these may partially be the product of amitriptyline’s downregulation of angiogenesis [309]. The degree to which sphingolipid metabolism is implicated in amitriptyline’s adverse cardio-ablative effects compared to its modulation of adrenergic signalling remains to be discerned.

#### 6.3.6. Desipramine

Desipramine is another TCA, like amitriptyline, that has been shown to mediate cardioprotective effects via inhibition of the Ac-SMase enzyme. In rabbit models of atherosclerosis, daily desipramine reduces plasma and vascular ceramide levels and reduces atherosclerotic plaque thickness, macrophage content, and MMP activity leading to greater plaque stability [300]. Evidence of unchanged cholesterol profiles further supports the theory that desipramine’s plaque-morphology-altering effects are independent of cholesterol synthesis and metabolism pathways [300]. This is corroborated by Jiang et al., who report that in db/db mice, desipramine improved HOMAIR and ameliorated endothelial dysfunction by increasing eNOS phosphorylation and NO bioavailability [310]. Ex vivo experiments on rat heart models of ischaemia and reperfusion, demonstrate desipramine reduces cardiac ceramide accumulation and myocardial infarct volumes, although not when combined with ischaemic preconditioning [311]. Ischaemic preconditioning appears to trigger protective ceramide signalling pathways through the conversion of ceramide to S1P which may then activate anti-apoptosis pathways [311].

Further cardioprotective effects have been reported in murine sepsis models in which desipramine promotes the maintenance of effective systolic and diastolic cardiac function during a systemic inflammatory insult [312]. Desipramine also alters gene expression in cardiac tissue to prevent sepsis-induced remodelling and cardiomyopathy [312]. There seems to be conflicting evidence regarding desipramine’s impact on ceramide synthesis with some studies demonstrating altered gene expression reflecting upregulation of de novo ceramide biosynthesis [300]. Further investigation of desipramine’s effects on ceramide biosynthesis gene expression is needed to further clarify the full mechanism of its clinical effects.

Finally, recent evidence suggests desipramine, amitriptyline, and fluoxetine may confer therapeutic benefits in recovering from acute ischaemic stroke. Inhibition of Ac-SMase by desipramine, amitriptyline, and fluoxetine reduced ceramide levels and upregulated angiogenesis resulting in increased in vivo neuronal survival in mice subjected to middle cerebral artery occlusion [313]. Cell culture studies point towards endothelial cell release of extracellular vesicles containing angiogenic proteins as the mechanism behind TCA-induced angiogenesis [313]. This recent discovery now needs studies to corroborate these findings and then examine the clinical effect in humans on these medications.

#### 6.3.7. N-Acetylcysteine

N-acetylcysteine is the prodrug of L-cysteine which in turn is the precursor to glutathione, a potent antioxidant. N-acetylcysteine is, therefore, able to quickly replenish glutathione stores and is best known for its role in the management of paracetamol overdose. Given the increasing awareness of the role ROS plays in cardiac pathology, N-acetylcysteine’s antioxidant properties may make it an ideal candidate for the treatment of cardiovascular disease.

Studies in Wistar rats show a range of therapeutic benefits across a spectrum of cardiovascular pathologies. Most recently, Hodun et al. report significant metabolic improvements as N-acetylcysteine reduced body weight, plasma glucose and insulin, and HOMAIR in rats on both low- and high-fat diets compared to controls [314]. The enhancement of insulin sensitivity is corroborated by demonstrably increased phosphorylation of Akt and GSK3β in response to insulin [314]. N-acetylcysteine also downregulated the expression of enzymes involved in de novo, salvage, and hydrolysis pathways resulting in reduced levels of deleterious ceramides in the plasma and myocardium and increased levels of the protective S1P in the same [314]. Other studies highlight cardiac glutathione depletion in post-MI hearts as a key oxidative mechanism for deteriorating cardiac function post-MI [315]. Replenishment of glutathione and its subsequent inhibition of N-SMase replenishes Bcl2 levels and inhibits Caspase 3 activation to thereby reduce cardiomyocyte cell death post-MI [315]. This results in reduced ventricular hydrogen peroxide and other lipid peroxidation product levels further ameliorating the pathological oxidative pathways upregulated in IHD [315].

There may also be a protective role for N-acetylcysteine in hypertension-induced cardiac hypertrophy. N-acetylcysteine also improves fractional shortening and reduces ventricular wall thickness, MMP activity, and myocardial collagen deposition in rat models of hypertension resulting in improved ventricular function [316]. TNFα utilises N-SMase to induce cell apoptosis when myocardial glutathione levels are low [317]. Glutathione inhibits N-SMase, so replenishment of myocardial glutathione stores also inhibits deleterious TNFα signalling [316]. Notably, N-acetylcysteine did not confer any reduction in systolic blood pressure in hypertensive rats [316]. Furthermore, whilst Adamy et al. do show improvement in both systolic and diastolic dysfunction and cardiac fibrosis post-MI, there was no significant change specifically in cardiac hypertrophy with N-acetylcysteine [315].

The extent of the mechanisms through which N-acetylcysteine ameliorates deleterious sphingolipid cardiac signalling is unclear. There is good evidence for the antioxidant properties of glutathione and so it may be that glutathione reduces ROS production which then reduces ROS-mediated intracellular processes, such as dysregulation of insulin signalling, instigation of myocardial tissue damage, and initiation of cell apoptosis. It’s unclear whether the downregulation of ceramide pathways are direct effects of glutathione or indirect effects as a result of reduced oxidative stress on intracellular signalling. It is well known, however, that glutathione directly inhibits N-SMase [318]. Further mechanistic studies to better understand the role of N-acetylcysteine are needed as are human trials designed with a priori cardiovascular outcomes. N-acetylcysteine is known to be a safe drug in humans and is increasingly undergoing in-human clinical trials with the aim of repurposing the antioxidant in well-established conditions. Its potential for use in cardiovascular disease, therefore, is significant.

#### 6.3.8. SPT Inhibitors

A number of rodent studies have examined the efficacy of an inhibitor of the SPT enzyme, known as myriocin [196,217,275,319,320,321,322,323,324]. Naturally, SPT inhibition reduces both plasma and tissue (myocardium and skeletal muscle) ceramide levels and, in some cases, was shown to reduce levels of other sphingolipid species as well [196,217,275,319,320,321,322,323,324]. Most SPT inhibitor studies report significantly improved serum cholesterol, triglyceride, and free fatty acid profiles [196,217,275,319,321,324]. In keeping with these effects, reductions in aortic root and brachiocephalic atherosclerotic plaque sizes suggest SPT inhibitors also prevent atherogenesis [196,321,323]. Two studies also demonstrated that SPT inhibition prevented pathological post-ischaemia myocardial remodelling leading to improved indices of left ventricular function [275,324].

SPT-inhibitor-mediated reduction in ceramide levels also has metabolic benefits. Some studies demonstrate that myriocin ameliorates clinical manifestations of insulin resistance such as peripheral glucose tolerance and upregulates insulin-stimulated Akt phosphorylation, thus preventing clinical transformation to frank T2DM in both genetic and diet-induced models of diabetes [217,319,320]. Myriocin reduces hepatic steatosis and enhances energy expenditure [319]. It also modifies adipose gene expression to downregulate the production of pro-inflammatory and prothrombotic messengers such as MCP-1 and PAI-1 [319]. Apart from Yang et al., in which a reduced rate of bodyweight gain is reported, SPT-inhibitor studies typically show no significant effect on food intake or body weight [196,217,275,319,320,321,322,323].

With a reasonably strong evidence base for cardiometabolic benefits, clinical trials of SPT inhibitors are the logical next step. One of the challenges, however, is the non-specificity of some SPT inhibitors that could lead to a wide range of off-target effects. Furthermore, as SPT is the critical first catalytic step in de novo sphingolipid synthesis, its inhibition would undoubtedly alter levels of sphingolipids that are vital to physiological function. Such challenges need to be addressed before SPT inhibitors can become a viable cardiovascular therapeutic.

#### 6.3.9. SphK Inhibitors

The evidence base for the use of SphK inhibitors in CVD is small but may be indicative of some potentially promising benefits in hypertension. Intraperitoneal selective SphK1 inhibitors improved endothelial function (possibly through reducing an inhibitory eNOS phosphorylation) and reduced cardiac hypertrophy in hypertensive mice [325]. Specific SphK2 inhibition in hypertensive rodents, on the other hand, was shown to significantly lower systolic blood pressure [326]. SphK inhibitors are a relatively new area of therapeutic interest in CVD, so clinical trials in humans will be needed before any conclusions pertaining to their efficacy can be drawn.

#### 6.3.10. UDP-Glucose Ceramide Glucosyltransferase Inhibitors

Two UGlu-CG inhibitors, miglustat and eliglustat, are available, licensed, and used in the management of lysosomal and glycogen storage disorders; typically Type 1 Gaucher’s disease, Niemann-Pick type C disease, and Pompe disease. In glycogen storage disorders, enzymatic defects result in the accumulation of glucosylceramide and, therefore, UGlu-CG inhibitors are used to limit this accumulation. Whilst these medications have been in use for approximately 20 and 9 years, respectively, they have not been employed in cardiovascular disease, chiefly due to limited supporting evidence thus far.

D-Threo-1-phenyl-2-decanoylamino-3-morpholino-1-propanol (D-PDMP) is another ceramide analogue and inhibits UGlu-CG (also known as glucosylceramide synthase) thereby ablating glucosylceramide synthesis. Several animal studies have demonstrated the benefits of D-PDMP in both ischaemic and hypertrophic cardiovascular disease [327,328,329,330]. Chaterjee et al. report D-PDMP improves atherosclerosis phenotypes in apolipoprotein E (ApoE) knockout mice, reducing lesion size, calcification, and lipid content at lower doses and completely preventing atherosclerosis at higher doses [327,329,330]. D-PDMP also seems to improve LDL, HDL and triglyceride levels also [327]. Later studies elucidate this further, showing that D-PDMP upregulates the expression of genes involved in cholesterol metabolism and efflux [329,330]. D-PDMP also improves cardiac phenotypes in mouse models of cardiac hypertrophy by reducing myocardial wall thickness, left ventricular mass, and fractional shortening [328,329]. Similarly, D-PDMP also effects changes to gene expression, downregulating the expression of heavy myosin chains implicated in cardiac hypertrophy as well as precursors to important hypertrophy biomarkers ANP and BNP [328,329].

Glycosphingolipids like glucosylceramide and lactosylceramide inflict pathological cardiac phenotypes via multiple pathways. Glucosylceramide may promote the production of ROS by uncoupling eNOS [301]. Lactosylceramide may promote the production of ROS via NADPH oxidase activation [327]. Both may also independently trigger signalling cascades leading to the activation of pathological transcription factors [330]. Inhibition of p42/44MAPK or p21ras may inhibit downstream activation of signalling kinases and transcription factors promoting cardiac hypertrophy and atherosclerosis [328,329]. The mammalian target of rapamycin complex 1 is a common target of both lysosomal cholesterol and D-PDMP, and its D-PDMP-mediated translocation or inhibition may be implicated in the upregulation of cholesterol efflux [330]. Mishra et al. have devised a biodegradable polymer capsule that facilitates controlled drug delivery, prolonged half-life, and significant clinical effect at a fraction of the standard trial dose, thus opening the door for in-human clinical trials [329].

#### 6.3.11. Dihydroceramide DES Inhibitors

Dihydroceramide DES is the enzyme that catalyses the final step of the de novo ceramide synthesis pathway, desaturating dihydroceramide into ceramide. Thus, inhibition of DES to disrupt the de novo synthesis of deleterious ceramide is a logical therapeutic approach. Fenretinide is a DES1 inhibitor and has produced mixed results in animal studies. Generally, evidence agrees that fenretinide reduces expression of retinol-binding protein 4 an adipokine thought to contribute to insulin resistance, and as a result, improved insulin sensitivity [218,329,330]. Though whereas Koh et al. and Bickman et al. report improvements in serum glucose and insulin, HOMAIR, and glucose tolerance, Thompson et al. report increased plasma glucose and reduced glucose tolerance [218,330]. Studies generally also agree that fenretinide reduces or prevents hepatic steatosis and the progression of non-alcoholic fatty liver disease [218,330].

Consensus is lacking, however, on fenretinide’s effects on body weight and circulating ceramide levels with different studies reporting both reduced and unchanged readings of each. Understandably, dihydroceramide levels are reported as being raised; however, whilst Koh et al. and Bickman et al. describe reduced measured ceramide levels, Thompson et al. report paradoxically increased ceramide levels as a result of compensatory activation of SMase enzymes, stimulated by fenretinide and mediated by retinoic acid [218,330]. Thompson et al. also report suppression of HMG-CoA reductase expression but paradoxically with no reduction in serum cholesterol and an increase in serum triglycerides. Koh et al. conversely report significant reductions in total cholesterol, triglycerides and free fatty acids [330]. Only Thompson et al. report on atherosclerotic outcomes and suggest that despite downregulation of genes associated with cardiovascular inflammation, remodelling, and fibrosis, LDL-R knockout mice display a worsening plaque burden with fenretinide, presumably in response to the compensatory increase in circulating plasma ceramides and triglycerides [218].

The studies highlighted here use different mouse models including Ob/Ob, LDL-R knockout, and unaltered C57Bl6 makes evaluation and judgement of the evidence difficult. Whilst fenretinide demonstrates clear improvements in metabolic health, it’s value in cardiovascular disease is inconclusive and further trials are needed to clarify this unknown.

## 7. Conclusions

In summary, sphingolipids play an important role in both ischaemic and non-ischaemic cardiovascular disease. The interactions between sphingolipids and the fundamental pathophysiological mechanisms for these conditions are complex. This complexity extends to the task of designing therapeutics against sphingolipids. Pharmacological targeting of sphingolipid metabolism and signalling remains an unmet challenge. Though the evidence suggests promising initial results in both novel and established pharmacological therapies targeting sphingolipids, more in-human trials measuring long-term clinical cardiovascular outcomes are needed to better gauge their real-world therapeutic potential.

## Figures and Tables

**Table 1:** A Table summarising the physiological and pathophysiological effects of sphingolipid species on the cardiovascular system. eNOS: Endothelial Nitric Oxide Synthase, NO: Nitric Oxide, VSMC: Vascular Smooth Muscle Cell, LDL: Low-Density Lipoprotein Cholesterol, Ox-LDL: Oxidised LDL, MCP-1: monocyte chemoattractant protein-1, IL-6: Interleukin 6, TNFα: Tumour Necrosis Factor Alpha, NOX: NADPH Oxidase, ROS: Reactive Oxygen Species, SMase: Sphingomyelinase, SPT: Serine Palmitoyltransferase, HOMAIR: Homeostatic Model Assessment of Insulin Resistance, BMI: Body Mass Index, T2DM: Type 2 Diabetes Mellitus, RANTES: Regulated on Activation, Normal T Cell Expressed and Secreted, ApoM: Apolipoprotein M, HDL: High-Density Lipoprotein Cholesterol, NF-κB: Nuclear Factor Kappa Beta, COX2: Cyclooxygenase 2, S1PR1-5: S1P Receptors 1-5, SphK: Sphingosine Kinase, IL-1β: Interleukin 1 Beta T1DM: Type 1 Diabetes Mellitus, MMP: Matrix Metalloproteinase, MIP-1β: Macrophage Inflammatory Protein 1 Beta, GM3: Monosialodihexosylganglioside, ICAM-1: Intercellular Adhesion Molecule 1.

Sphingolipid Species	Effect on Cardiovascular Physiology and Pathophysiology						
	Atherosclerosis	Endothelial Dysfunction	Dyslipidaemia	Inflammation and Immune Modulation	Oxidative Stress	Tissue Death/ Remodelling	Insulin Resistance
Ceramide	- Increased levels in atherosclerotic plaques. - Increased levels in acute ischaemic and haemorrhagic stroke brain tissue as well as in the reperfusion period. - Accumulates in the ischaemic core and peri-ischaemic brain regions.	- Induces eNOS uncoupling and reduced NO bioavailability - Promotes vasoconstriction and increased vascular permeability. - Induces mitochondrial permeability which triggers caspase activation and endothelial cell apoptosis. - Induces VSMC apoptosis and contributes to aneurysm formation. - Induces cerebral vasoconstriction and leukocyte adhesion, predisposing to herald bleeds. Increased levels associated with increased systolic blood pressure.	- Positively correlates with total cholesterol and LDL. - Conversion of LDL membrane Sphingomyelin to Ceramide leads to hydrophobic LDL particles and increased LDL aggregation. - Stabilises endothelial membrane rafts containing Lox-1, Cavin-1, and Caveolin-1, responsible for Ox-LDL internalisation.	- Increased plaque levels associated with raised MCP-1 and IL-6. - Endothelial cell Ceramide generation enhanced by TNFα. - Increased intracellular Ceramide associated with increased adhesion molecule expression in monocytes, and lead to increased vascular adhesion, OxLDL internalisation and foam cell formation. - Promote myocardial immune cell infiltration and inflammation leading to diminished cardiac function.	- Upregulates NOX and eNOS-mediated ROS production. - Enhances mitochondrial ROS production through disruption of the electron transport chain. - ROS production leads to cell damage and apoptosis. - Ceramide upregulated by ROS-mediated SMase activation.	- Hypoxia upregulates SPT activity, increases Ceramide synthesis, increasing apoptosis. - Increased C16 Ceramide associated with adverse cardiac remodelling. - De novo synthesis upregulated in failing cardiac tissue. Accumulation leads to cardiomyocyte apoptosis.	- Increased in obesity and positively correlated with waist circumference, BMI and HOMAIR. - C24 Ceramide replacement my be metabolically beneficial, increasing weight loss, insulin sensitivity, glucose tolerance, and FFA oxidation. - Directly and indirectly inhibit phosphorylation of Akt thereby impairing cellular insulin signalling, causing insulin resistance. - Accumulates in the pancreas in T2DM.
Dihydroceramide	- Increased levels in advanced atherosclerotic plaques.			- Increased plaque levels associated with raised MCP-1 and IL-6.			
Sphingomyelin	- Increased levels in advanced atherosclerotic plaques.		- Reduces intestinal cholesterol absorption, ameliorating hyperlipidaemia.	- Increased plaque levels associated with raised MCP-1 and IL-6.			
Sphingosine 1-Phosphate		- Can induce vasoconstriction and increased vascular permeability at supraphysiological concentrations. - Reduces eNOS activity and NO bioavailability. - Triggers assembly of endothelial adherens junctions and stabilisation of existing junctions ensuring preservation of endothelial barrier function. - Promotes endothelial cell proliferation and survival.	- Levels positively correlated with total cholesterol and LDL.	- Increased plaque levels associated with raised TNFα and RANTES. - Upregulates lymphocyte activation, adhesion molecule expression, and intimal migration. - Upregulates endothelial adhesion molecule expression. - Binds to ApoM-HDL in the circulation and, through this association, inhibits TNFα-induced NF-κB signalling and endothelial adhesion molecule expression. - Can upregulate endothelial COX2 expression and subsequent prostaglandin E2 secretion through S1PR3 signalling. - Associated with reduced myocardial inflammation.		- Associated with lower prevalence of aneurysmal disease. - Associated with reduced myocardial remodelling. - Promotes cardiac fibrosis (as a result of overexpression of SphK and S1PR1&3). - HDL-S1P reduces IL-1β-induced β-islet apoptosis.	- S1PR2 (responsible for atherogenic effects) is upregulated in hyperglycaemia. - Levels increased in obesity and positively correlated with waist circumference, BMI, and HOMAIR. - S1PR activation upregulates Akt phosphorylation, improving insulin signalling and enhancing β-islet survival in T1DM.
Ceramide-1-Phosphate		- May promote neointima formation and endothelial repair.		- Inhibits cigarette smoke-induced inflammation by downregulating TNFα, 1L-1β, and IL-6 expression. - Has paradoxically also been shown to activate TNFα, IL-6, and phospholipase A2 to promote prostaglandin synthesis.	- Activates MMP2 and MMP9 leading to aortic matrix destruction.		
Glucosylceramide	- Accumulates in the intima of atherosclerotic plaques. - Increased levels in advanced atherosclerotic plaques. - Purported anticoagulant effect may be protective. - Levels of complex gangliosides diminish and simple gangliosides increase in brain tissue subjected to hypoxic ischaemia.	- Induces VSMC apoptosis.		- Increased plaque levels associated with raised MCP-1 and IL-6. - Stimulates VSMC MIP-1β, TNFα, MCP-1, and RANTES secretion. - Plaque levels positively correlated with plaque CD68 macrophage levels.		- Conversion of simple to complex gangliosides in ischaemic tissue leads to increased tissue damage, increased neuronal death, and diminished neurological recovery.	- Directly act on the insulin receptor to impair cellular insulin signalling, causing insulin resistance. - GM3 is an especially potent inhibitor of inulin signalling.
Galactosylceramide	- Levels of complex gangliosides diminish and simple gangliosides increase in brain tissue subjected to hypoxic ischaemia.			- Plaque levels positively correlated with plaque CD68 macrophage levels.		- Conversion of simple to complex gangliosides in ischaemic tissue leads to increased tissue damage, increased neuronal death, and diminished neurological recovery.	
Lactosylceramide	- Accumulates in the intima of atherosclerotic plaques. - Increased levels in advanced atherosclerotic plaques. - Promotes VSMC proliferation, aiding fibrous cap formation. - Levels of complex gangliosides diminish and simple gangliosides increase in brain tissue subjected to hypoxic ischaemia.			- Increased plaque levels associated with raised MCP-1 and IL-6. - Plaque levels positively correlated with plaque CD68 macrophage levels. - Upregulates endothelial cell expression of NF-κB and ICAM-1.		- Conversion of simple to complex gangliosides in ischaemic tissue leads to increased tissue damage, increased neuronal death, and diminished neurological recovery.	

## Data Availability

This is a review of published literature, with no new data generated.

## Abbreviations

Ac-CDase:	Acid Ceramidase
Ac-SMase:	Acid Sphingomyelinase
AF:	Atrial Fibrillation
Ak-CDase:	Alkaline Ceramidase
Ak-SMase:	Alkaline Sphingomyelinase
AMPK:	AMP-activated Protein Kinase
ANP:	Atrial Natriuretic Peptide
ApoE:	Apolipoprotein E
ApoM:	Apolipoprotein M
BCL-2:	B-cell Lymphoma 2
BMI:	Body Mass Index
BNP:	Brain Natriuretic Peptide
C1P:	Ceramide-1-Phosphate
C1PP:	C1P phosphatase
CDase:	Ceramidase
CerK:	Ceramide Kinase
CerS:	Ceramide Synthase
CNS:	Central Nervous Systems
CoA:	Co-enzyme A
COX2:	Cyclooxygenase 2
CVD:	Cardiovascular Disease
DES1:	Desaturase 1
D-PDMP:	D-Threo-1-phenyl-2-decanoylamino-3-morpholino-1-propanol
eNOS:	Endothelial Nitric Oxide Synthase
ER:	Endoplasmic Reticulum
FFA:	Free Fatty Acids
GLP-1R:	Glucagon-like Peptide-1 Receptor
GM1:	Monosialotetrahexosylganglioside
GM3:	Monosialodihexosylganglioside
HCASMCs:	Human Coronary Artery Smooth Muscle Cells
HDL:	High-Density Lipoprotein Cholesterol
HF:	Heart Failure
HFpEF:	Heart Failure with Preserved Ejection Fraction
HFrEF:	Heart Failure with Reduced Ejection Fraction
HOA:	High Oleic Acid Diet
HOMAIR:	Homeostatic Model Assessment of Insulin Resistance
HPA:	High Palmitic Acid Diet
HUVECs:	Human Umbilical Vein Endothelial Cells
IHD:	Ischaemic Heart Disease
IL-1β:	Interleukin-1β
IL-6:	Interleukin 6
JNK:	c-Jun N-terminal Kinase
LDL:	Low-Density Lipoprotein Cholesterol
LDL-R:	LDL receptor
LVEF:	Left Ventricular Ejection Fraction
MACE:	Major Adverse Cardiovascular Events
MCP-1:	monocyte chemoattractant protein-1
MI:	Myocardial Infarction
MMPs:	Matrix Metalloproteinases
MPLs:	Milk Polar Lipids
MSM:	Milk Sphingomyelin
NADPH:	Nicotinamide Adenine Dinucleotide Phosphate
NF-κB:	Nuclear Factor Kappa Beta
NKT:	Natural Killer T Cell
NO:	Nitric Oxide
NOX:	NADPH Oxidase
N-CDase:	Neutral Ceramidase
N-SMase:	Neutral Sphingomyelinase
Ox-LDL:	Oxidised LDL
PAI-1:	Plasminogen Activator Inhibitor 1
PCSK9:	Protein Convertase Subtilisin/Kexin Type 9
PI3K:	Phosphatidylinositol 3-Kinase
PKC:	Protein Kinase C
PNS:	Peripheral Nervous System
PP2A:	Protein Phosphatase 2A
RANTES:	Regulated on Activation, Normal T Cell Expressed and Secreted
RCT:	Randomised Control Trial
ROS:	Reactive Oxygen Species
RYGB:	Roux-en-Y Gastric Bypass
S1P:	Sphingosine-1-Phosphate
S1PP:	S1P Phosphatase
S1PR1-5:	S1P Receptors 1-5
SFA:	Saturated Fatty Acid
SGLT-2:	Sodium Glucose Transporter 2
SMase:	Sphingomyelinase
SMS:	Sphingomyelin synthase
SphK:	Sphingosine Kinase
SPL:	Sphingosine-1-Phosphate Lyase
SPT:	Serine Palmitoyltransferase
T1DM:	Type 1 Diabetes Mellitus
TACE:	TNFα-Converting Enzyme
TCA:	Tri-cyclic Antidepressant
TLR4:	Toll-like Receptor 4
TNFα:	Tumour Necrosis Factor Alpha
UDP:	Uridine Diphosphate
UFA:	Unsaturated Fatty Acid
UGal-CG:	UDP-Galactose Ceramide Galactosyltransferase
UGlu-CG:	UDP-Glucose Ceramide Glucosyltransferase
VEGFR2:	Vascular Endothelial Growth Factor Receptor 2
